# The intron in centromeric noncoding RNA facilitates RNAi-mediated formation of heterochromatin

**DOI:** 10.1371/journal.pgen.1006606

**Published:** 2017-02-23

**Authors:** Masatoshi Mutazono, Misato Morita, Chihiro Tsukahara, Madoka Chinen, Shiori Nishioka, Tatsuhiro Yumikake, Kohei Dohke, Misuzu Sakamoto, Takashi Ideue, Jun-ichi Nakayama, Kojiro Ishii, Tokio Tani

**Affiliations:** 1 Department of Biological Sciences, Graduate School of Science and Technology, Kumamoto University, Kurokami, Chuo-ku, Kumamoto, Japan; 2 Laboratory of Chromosome Function and Regulation, Graduate School of Frontier Biosciences, Osaka University, Osaka, Japan; 3 Graduate School of Natural Sciences, Nagoya City University, Nagoya, Japan; 4 Division of Chromatin Regulation, National Institute for Basic Biology, Okazaki, Japan; University of California San Francisco, UNITED STATES

## Abstract

In fission yeast, the formation of centromeric heterochromatin is induced through the RNA interference (RNAi)-mediated pathway. Some pre-mRNA splicing mutants (*prp*) exhibit defective formation of centromeric heterochromatin, suggesting that splicing factors play roles in the formation of heterochromatin, or alternatively that the defect is caused by impaired splicing of pre-mRNAs encoding RNAi factors. Herein, we demonstrate that the splicing factor spPrp16p is enriched at the centromere, and associates with Cid12p (a factor in the RNAi pathway) and the intron-containing *dg* ncRNA. Interestingly, removal of the *dg* intron, mutations of its splice sites, or replacement of the *dg* intron with an euchromatic intron significantly decreased H3K9 dimethylation. We also revealed that splicing of *dg* ncRNA is repressed in cells and its repression depends on the distance from the transcription start site to the intron. Inefficient splicing was also observed in other intron-containing centromeric ncRNAs, *dh* and antisense *dg*, and splicing of antisense *dg* ncRNA was repressed in the presence of the RNAi factors. Our results suggest that the introns retained in centromeric ncRNAs work as facilitators, co-operating with splicing factors assembled on the intron and serving as a platform for the recruitment of RNAi factors, in the formation of centromeric heterochromatin.

## Author summary

Formation of centromeric heterochromatin is required for correct segregation of sister chromatids during mitosis. In fission yeast, formation of heterochromatin at centromeres is performed through the RNA interference (RNAi) system, which involves processing of noncoding RNAs transcribed from the centromeres. We found that the intron in the centromeric *dg* ncRNAs facilitates formation of centromeric heterochromatin in fission yeast. We showed that the splicing factor spPrp16p associates with the RNAi factor and intron-containing *dg* ncRNA. Removal of or mutations in the *dg* intron significantly decreased H3K9 dimethylation, suggesting that the intron and associated splicing factors serve as a platform for recruitment of RNAi factors. Inefficient splicing is a hallmark of intron-containing centromeric ncRNAs. Such repression of splicing seems to be important for facilitation of heterochromatin formation. Introns in euchromatic regions are removed by splicing to generate functional RNAs, whereas centromeric introns are retained in ncRNAs by splicing repression and play roles in gene silencing. Our findings shed light on the novel roles of introns in epigenetic regulation of gene expression and heterochromatin formation.

## Introduction

Chromosome segregation is a fundamental process in the transmission of genetic information to the daughter cells in eukaryotic cells. During mitosis, formation of heterochromatin at centromeres is essential for correct segregation of chromosomes, because centromeric heterochromatin provides an environment that promotes the assembly of the kinetochore, the protein complex that serves as an attachment site for microtubules [1].

The RNA interference (RNAi) machinery in the fission yeast *Schizosaccharomyces pombe* was previously reported to be involved in the formation of centromeric heterochromatin [2–4]. Noncoding RNAs (ncRNAs) are transcribed from centromeres, which consist of repetitive sequences named *dg* and *dh*, by RNA polymerase II. Double-stranded RNAs (dsRNAs) are synthesized from transcribed ncRNAs by RNA-directed RNA polymerase (Rdp1p), and are then processed into small interfering RNAs (siRNAs) by Dicer. siRNAs then associate with Ago1p to form the RNAi-induced Transcriptional Silencing (RITS) complex with Chp1p and Tas3p [2], and function as guide molecules that direct the RITS complex to nascent ncRNAs through base pairing interactions. The RITS complex recruits the CLRC complex, which contains the methyltransferase Clr4p, to the pericentromere region, where it promotes the dimethylation of histone H3 lysine 9 (H3K9me2). Dimethylation of H3K9 creates a binding site for Swi6p, a heterochromatin protein 1 (HP1) homologue, to form heterochromatin.

Recent studies reported that mutations in specific splicing factors, such as Cwf10p (Complexed with Cdc5) and Prp10p (pre-mRNA processing 10), and spliceosomal U4 small nuclear RNA (snRNA) decreased the generation of siRNAs from centromeric ncRNAs, resulting in the defective formation of heterochromatin at centromeres [5, 6]. These findings suggest that the splicing machinery is involved in RNAi-mediated heterochromatic gene silencing at centromeres. Regarding the role of the splicing machinery in RNAi-directed heterochromatin formation, we found an mRNA-type intron in centromeric *dg* ncRNA and proposed a model in which the spliceosome (or sub-spliceosome consisting of a part of the splicing factors) that assembles on the intron in centromeric ncRNA serves as a platform for the binding of RNAi factors to centromeric ncRNAs, thereby promoting RNAi-mediated formation of centromeric heterochromatin [6]. On the other hand, it was recently reported that proper splicing of pre-mRNAs encoding RNAi factors is necessary for heterochromatin formation at the pericentromere region, suggesting that defective formation of centromeric heterochromatin in *prp* splicing mutants is a secondary effect of defective splicing of pre-mRNAs encoding RNAi factors [7].

In the present study, we showed that the splicing factor encoded by the *prp16*^*+*^ gene is enriched at the centromeres and interacts with Cid12p, a component of the RNA-dependent RNA polymerase complex (RDRC), in *S*. *pombe*. We also showed that the intron in *dg* centromeric ncRNA plays an important role in promoting the RNAi-mediated formation of centromeric heterochromatin. Our results indicate that the splicing machinery and intron in centromeric ncRNA play intrinsic roles in the formation of centromeric heterochromatin.

## Results

### *prp14* exhibits defects in formation of centromeric heterochromatin

*prp14* was isolated as a pre-mRNA splicing mutant that accumulates pre-mRNAs at non-permissive temperatures in the fission yeast *Schizosaccharomyces pombe* [8]. It exhibits cold sensitivity (22°C) and temperature sensitivity (37°C) for growth, and grows slowly at the permissive temperature of 33°C [8]. Complementation cloning of the *prp14*^+^ gene revealed that the mutation in *prp14* resides in SPBC1711.17, which encodes an ATP-dependent RNA helicase homologous to *Saccharomyces cerevisiae* and human Prp16p (See S1 Text and S1 Fig), indicating that the *prp14* mutation is a *prp16* allele. Hereafter, we refer to *prp14* and its wild-type product as *prp16* and spPrp16p, respectively.

**Fig 1:**
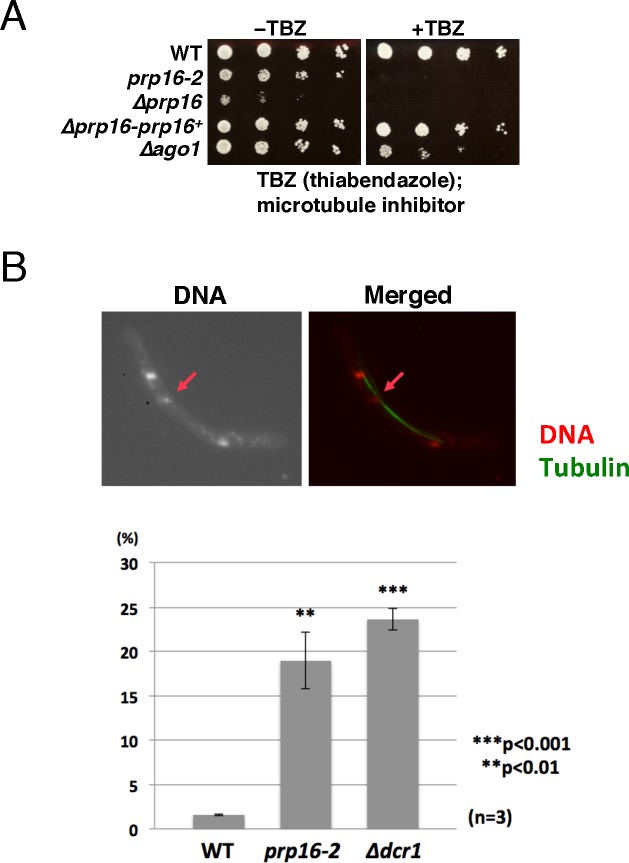
*prp16* has a defect in chromosome segregation. (A) *prp16-2* is hypersensitive to TBZ. Serially diluted cells indicated on the left of the panel were spotted on MMAU plates without TBZ (-TBZ) or with 10 μg/ml TBZ (+TBZ), and then incubated at 33°C for 5 days. *Δago1* was spotted as a control; this strain has a defect in RNAi-induced formation of centromeric heterochromatin. (B) *prp16-2* yields lagging chromosomes. Cells were double-stained with DAPI (red in the merged image) and antibody against tubulin (TAT1, green). Arrows indicate the lagging chromosome. The graph under the pictures shows the frequency of lagging chromosomes in late-anaphase cells in 972 (WT), *prp16-2*, or *Δdcr1*. Cells from three independent cultures at 33°C were analyzed. More than 50 late-anaphase cells were assessed for lagging chromosomes in each culture.

Some splicing mutants are defective in the RNAi-mediated formation of centromeric heterochromatin in *S*. *pombe* [5, 6], and we proposed a model in which the spliceosome or sub-spliceosome forms a platform for the recruitment of RNAi factors to ncRNAs transcribed from the centromere [6]. We found that *prp16-2* was hypersensitive to the microtubule-destabilizing drug thiabendazole (TBZ) (Fig 1A) and accumulated unprocessed *dg* ncRNA transcribed from centromeres [6], suggesting that *prp16* has defects in the attachment of microtubules to kinetochores and formation of centromeric heterochromatin.

**Fig 2:**
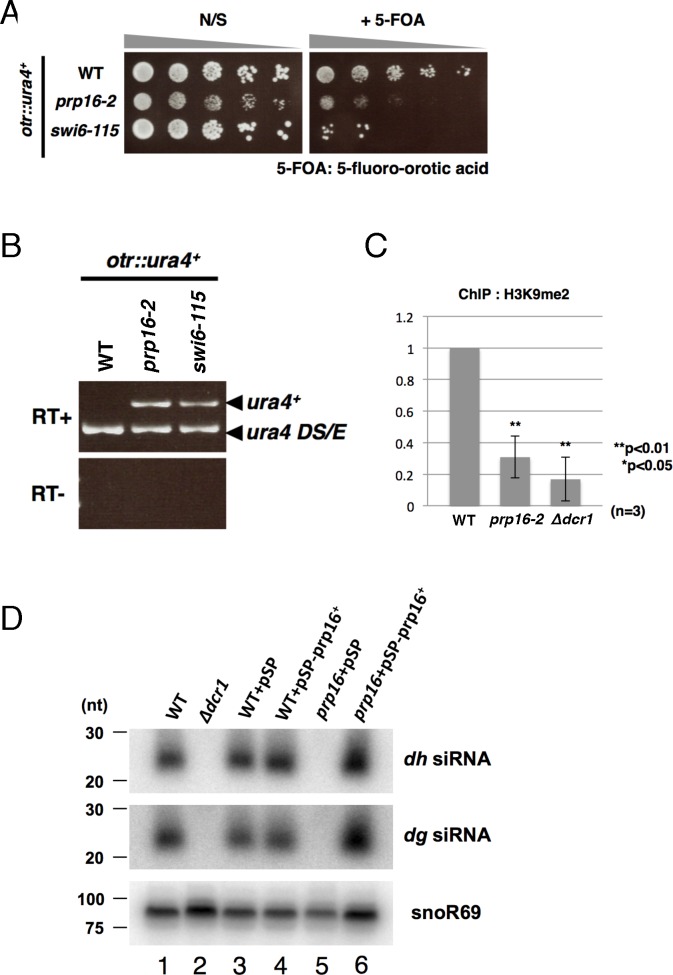
*prp16-2* exhibits defective formation of centromeric heterochromatin. (A) Serially diluted cells harboring the *ura4*^+^ gene inserted in the *otr1* region were spotted on plates containing 1 mg/ml 5-fluoroorotic acid (FOA). *prp16-2* and *swi6* mutants with the *ura4*^+^ gene were sensitive to 5’-FOA, indicating expression of the *ura4*^+^ marker gene inserted in the centromere region. The site of the *ura4*^+^ insertion is shown schematically in S2 Fig. (B) Expression of the *ura4*^+^ gene inserted in the centromere region in *prp16-2* and *swi6* was confirmed by RT-PCR. *Ura4DS/E* indicates a band derived from the authentic *ura4*^+^ gene, which harbors a 269 bp deletion. (C) ChIP analysis of H3K9me2 at the *dg* region was performed with the indicated strains using the anti-H3K9me2 antibody. *Δdcr1* is a mutant deleting the *dcr1*^+^ gene essential for the RNAi pathway. (D) Northern blot analysis of siRNA derived from centromeric *dg* ncRNA revealed a reduction in the amount of centromeric siRNAs in *prp16-2*. SnoR69 RNA was used as a loading control. Lane 1: HM123 (wild-type); 2: *Δdcr1*; 3: HM123 transformed with pSP1; 4: HM123 transformed with pSP1-*prp16*^+^; 5: *prp16-2* transformed with pSP1; 6: *prp16-2* transformed with pSP1-*prp16*^+^.

We further characterized *prp16* in terms of the relationship between the splicing machinery and centromeric gene silencing. As shown in Fig 1B, the *prp16-2* mutant exhibited a high incidence of lagging chromosomes during mitosis, consistent with defective attachment of microtubules to kinetochores. To examine the integrity of pericentromeric heterochromatin, which is required for the proper assembly of kinetochores, we constructed *prp16-2* with the *ura4*^*+*^ marker gene in the *otr1* region of centromere 1 (S2 Fig), and then serially spotted the resultant cells onto plates with or without 5-fluoroorotic acid (5-FOA). As shown in Fig 2A, *prp16-2* cells harboring the inserted *ura4*^+^ gene were highly sensitive to 5-FOA, similar to the *swi6* heterochromatin protein mutant, suggesting that, in these mutants, the *ura4*^+^ gene was expressed when inserted in centromeric regions. By contrast, a wild-type strain harboring the *ura4*^+^ gene inserted into the *otr1* region grew well on 5-FOA plates. RT-PCR analysis also revealed that the *ura4*^+^ gene inserted into the *otr1* region was expressed as in a *swi6* mutant harboring the same insertion (Fig 2B).

To further demonstrate the impaired formation of centromeric heterochromatin in *prp16*, we performed a chromatin immunoprecipitation (ChIP) analysis using an anti-H3K9 dimethylation (H3K9me2) antibody. As shown in Fig 2C, the *prp16* mutation decreased the H3K9me2 level in the pericentromeric region to a level observed in *Δdcr1*, indicating defective formation of pericentromeric heterochromatin in *prp16*. In addition, northern blot analysis revealed that the amount of centromeric siRNAs derived from *dg* ncRNA was markedly reduced in *prp16-2* (Fig 2D, lane 5), consistent with our previous report showing that *prp16* accumulates unprocessed *dg* ncRNA [6]. These results suggested that processing of centromeric ncRNAs is defective in *prp16*. Furthermore, expression of the wild-type *prp16*^+^ gene rescued defective production of centromeric siRNAs, indicating that the *prp16*^+^ gene is responsible for formation of centromeric heterochromatin (Fig 2D, lane 6).

### spPrp16p is enriched at the centromere in *S*. *pombe*

Because *prp16* was isolated in the screen for defective pre-mRNA splicing at the non-permissive temperature, the impaired formation of centromeric heterochromatin may have been caused by defects in splicing of pre-mRNAs encoding factors involved in RNAi-mediated formation of centromeric heterochromatin, as suggested by Kallgren *et al*. [7]. A RT-PCR analysis of splicing defects revealed that the *prp16-2* mutation impaired splicing of *ago1*^*+*^, *sir2*^*+*^, *hrr1*^*+*^, *arb2*^*+*^, *ers1*^*+*^, and *dsh1*^*+*^ pre-mRNAs at the temperatures at which analyses on heterochromatin formation were conducted (S3 Fig). This result suggests that the observed defects in the formation of centromeric heterochromatin in *prp16* are potentially due to splicing defects. On the other hand, *prp13-1*, which has defects in the formation of pericentromeric heterochromatin [6], similar to *prp16*, did not exhibit defects in pre-mRNA splicing for the RNAi factors analyzed, although very weak splicing defects were observed for Ago1p and Arb2p (S3 Fig, lanes *prp13-1*). In *prp13-1*, mature mRNAs encoding the RNAi factors were produced at levels almost equal to those in wild-type cells at all temperatures tested, suggesting that the defects observed in centromeric gene silencing in the *prp* mutants cannot be simply attributed to secondary effects of splicing defects.

**Fig 3:**
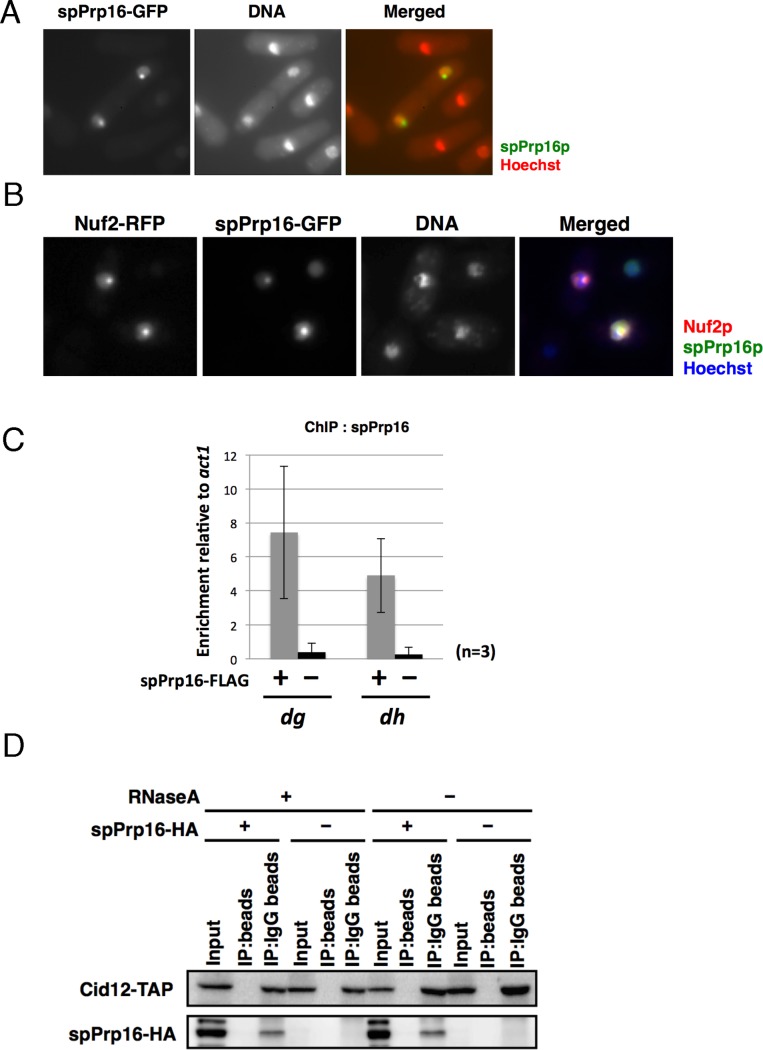
spPrp16p associates with RNAi factors. (A) spPrp16-GFP expressed from the multicopy plasmid exhibited a dot-like signal in the nucleus, in addition to diffuse nuclear distribution. Cells were cultured at 33°C and counterstained with Hoechst 33342. In the merged image, green and red indicate spPrp16p-GFP and Hoechst 33342 (DNA), respectively. (B) Enrichment of spPrp16p at the centromere region. Dot-like signals of spPrp16-GFP expressed from the plasmid and Nuf2-RFP, a kinetochore protein, expressed from its endogenous locus were colocalized in the nucleus, suggesting that spPrp16p is enriched at the kinetochore or centromere when overexpressed. Of 161 cells exhibiting co-expression of spPrp16-GFP and Nuf2-RFP, 42 cells (26%) showed dot-like signals of spPrp16-GFP and all of them colocalized with the Nuf2-RFP dot signals. Cells were stained with Hoechst 33342 before observations. (C) ChIP analysis of spPrp16-FLAG expressed from the endogenous locus at the *dg* and *dh* repeats. Enrichment of spPrp16-FLAG was calculated relative to the actin gene, which contains no intron. Cells with (+) or without (-) the plasmid expressing FLAG-tagged spPrp16p were used in these analyses. (D) RNA-independent interaction between spPrp16-HA and Cid12-TAP. A co-immunoprecipitation assay was performed using samples treated with (+) or without (-) RNase A. Cid12-TAP was immunoprecipitated using IgG–Sepharose beads (IP: IgG beads). Sepharose beads with no antibody were used as negative controls for immunoprecipitation. Extracts were prepared from cells expressing spPrp16p-HA and Cid12-TAP (spPrp16-HA: +) or only Cid12-TAP (spPrp16-HA: -). Western blot analysis was performed with an anti–protein A antibody (Cid12-TAP) or anti-HA antibody (spPrp16-HA).

Interestingly, spPrp16-GFP was detected in the nucleus as a dot-like signal with a diffuse nuclear distribution when it was over-expressed from a multicopy plasmid pSP1 (Fig 3A), but not when it was expressed from the endogenous locus (S4A Fig). Because *prp16* exhibited a defect in formation of centromeric heterochromatin, we speculated that the nuclear site of the dot-like signal of overexpressed spPrp16-GFP corresponded to centromeres or kinetochores. To test this idea, we co-expressed spPrp16-GFP and Nuf2-RFP, a kinetochore protein tagged with RFP, and observed their localization. The dot-like signal of over-expressed spPrp16-GFP was colocalized with the Nuf2-RFP signal, suggesting that spPrp16p was enriched at the centromere (or kinetochore) when over-expressed (Fig 3B). In addition, ChIP analysis using a strain expressing FLAG-tagged spPrp16p from the endogenous locus revealed that spPrp16p was enriched at the *dg* and *dh* repeats in the pericentromere region (Fig 3C). These results suggest that spPrp16p plays some roles at the centromeres, in addition to its function as an RNA helicase in the splicing reaction [9].

### spPrp16p interacts with Cid12p, a component of RDRC

To determine the role of spPrp16p in the formation of centromeric heterochromatin, we examined the physical interactions between spPrp16p and components of the RNAi pathway. To this end, we performed a co-immunoprecipitation assay using a strain expressing spPrp16p-HA and Cid12p-TAP or Chp1p-FLAG. Cid12p is a poly(A) polymerase family member protein that is a component of the RDRC, which binds centromeric ncRNAs to initiate dsRNA synthesis, and Chp1p is a component of the RITS complex that contains Ago1p [4, 10]. Although we were unable to detect an interaction between spPrp16p-HA and Chp1p-FLAG under the conditions tested (S4B Fig), we did detect spPrp16p-HA in the precipitate of Cid12-TAP, suggesting that spPrp16p interacts with Cid12p (Fig 3D, lanes RNase A-).

To determine whether the interaction between spPrp16p and Cid12p is RNA-dependent, we treated samples with RNase A and tested the interaction by co-immunoprecipitation. Treatment of samples with RNase A completely degraded *dg* RNAs (S4C Fig). As shown in Fig 3D (lanes marked as RNase A “+”), RNase treatment did not affect co-immunoprecipitation of Cid12p with spPrp16p, indicating that the association of spPrp16p with Cid12p is not mediated through RNA.

### The intron in *dg* ncRNA facilitates the formation of heterochromatin

**Fig 4:**
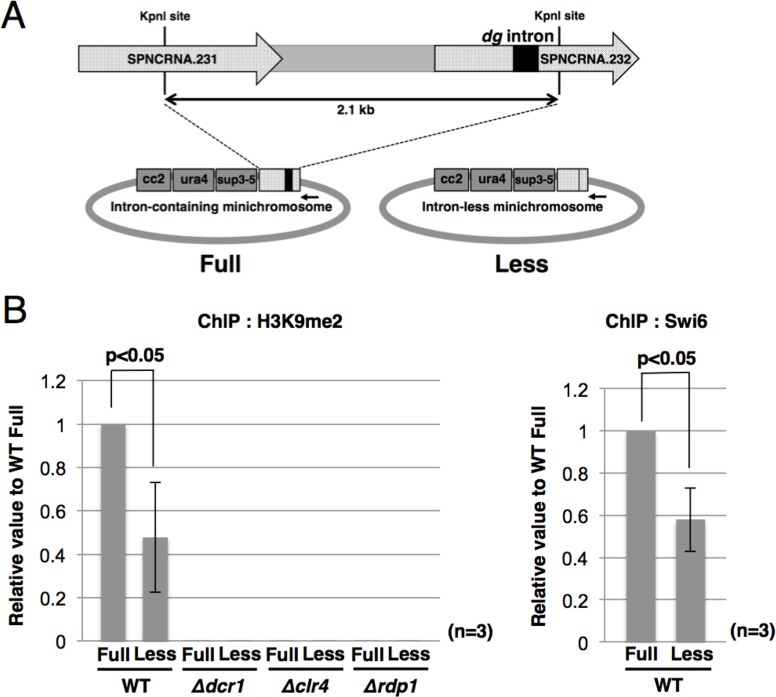
Removal of the intron from the *dg* transcript impairs the formation of heterochromatin. (A) Schematic representation of the minichromosomes. The intron-containing minichromosome (Full; pH-cc2) [11, 30] contains the cc2 (central domain) region, *ura4*^*+*^ gene, *sup3-e* gene, and a 2.1 kb *Kpn*I–*Kpn*I fragment derived from the *otr dg* region. The intron-less minichromosome (Less) lacks the *dg* intron. The arrow indicates the position of the minichromosome-specific reverse primer. (B) ChIP analyses of H3K9me2 (left panel) and Swi6p (right panel) at the *dg* sequences on the minichromosomes in the indicated strains. Quantitative PCR was performed using minichromosome-specific primers.

The physical association of spPrp16p with Cid12p supports the model proposed in our previous study, in which the spliceosome or sub-spliceosome assembled on the intron serves as a platform for recruiting RNAi factors [6]. To test the platform model further, we next investigated whether removal of the intron in *dg* ncRNA affects heterochromatin formation at the centromere using a minichromosome with or without the *dg* intron (Fig 4A). This minichromosome contains a central core sequence (cc2) and the part of the *dg* region necessary for centromere functions [11]. Interestingly, ChIP analyses of H3K9me2 and Swi6p heterochromatin protein using minichromosome-specific primers revealed that H3K9me2 and Swi6p levels on the *dg* region in the intron-less minichromosome (Less) were clearly lower than those in the intron-containing minichromosome (Full) (Fig 4B). In a strain harboring deletions of the *dcr1*^*+*^, *clr4*^*+*^, or *rdp1*^*+*^ genes, which are essential for the RNAi pathway, the level of H3K9me2 in the minichromosome was markedly reduced, demonstrating that dimethylation of H3K9 on the minichromosome is also mediated by the RNAi-mediated pathway. These results suggest that the *dg* intron facilitates the formation of heterochromatin.

**Fig 5:**
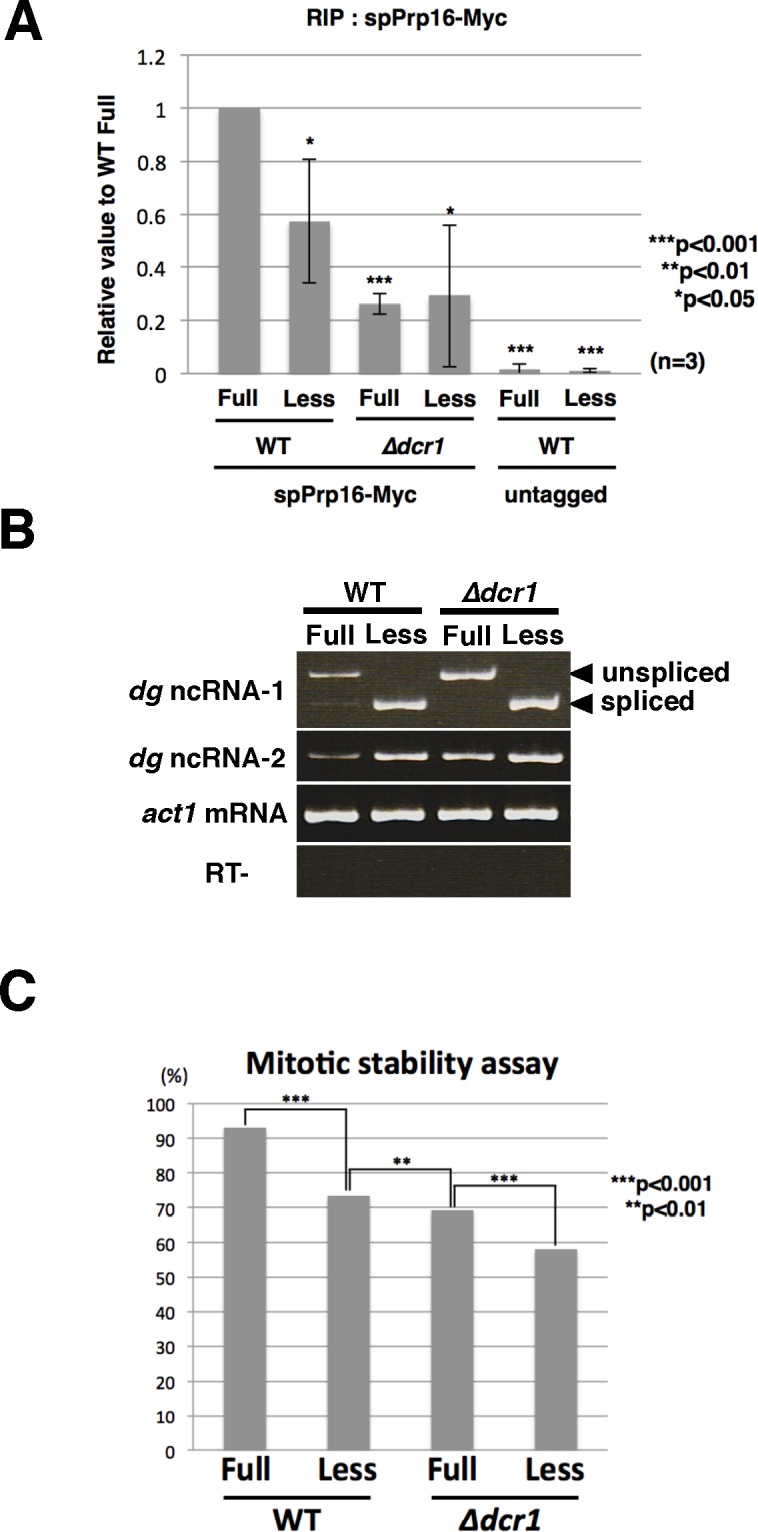
RIP and mitotic stability analysis. (A) Intron-less *dg* ncRNA bound spPrp16p less efficiently. RNA immunoprecipitation (RIP) analysis was carried out using the anti-Myc antibody and extracts prepared from wild-type or *Δdcr1* cells possessing the intron-containing (Full) or intron-less (Less) minichromosome. spPrp16-Myc was expressed from the endogenous locus. qRT-PCR was performed using minichromosome-specific primers. Relative values were calculated as a ratio of IP/WCE. spPrp16p without the Myc-tag (untagged) was used as a negative control. (B) Amounts of steady-state *dg* ncRNA expressed from the minichromosome were analyzed by RT-PCR. Reverse transcription was performed using minichromosome-specific primers, and PCR was performed using primers that amplify the region spanning the intron (*dg* ncRNA-1) or region downstream of the intron (*dg* ncRNA-2). *act1* mRNA was also detected as a loading control (*act1* mRNA). “RT-” represents the reaction without reverse transcriptase. The positions of unspliced and spliced *dg* ncRNAs are indicated on the right of the panel. The amount of intron-less *dg* transcripts (Less) was larger than the amount of intron-containing *dg* transcripts (Full). In cells lacking the *dcr1*^+^ gene (*Δdcr1*), the amount of the intron-containing *dg* transcripts was elevated. (C) The mitotic stability assay of the minichromosome with (Full) or without (Less) the *dg* intron in wild-type or *Δdcr1* cells. The p-value was calculated by a two-sample test for the equality of proportions with continuity corrections.

We also tested the association of spPrp16p with *dg* ncRNA with or without the intron (Fig 5A). RNA immunoprecipitation (RIP) analysis using minichromosome-specific primers revealed that the amount of intron-less *dg* ncRNA bound to spPrp16p-Myc was lower than that of intron-containing *dg* ncRNA (Fig 5A). Gel electrophoresis of RT-PCR products from the precipitates indicated that spPrp16p bound both unspliced and spliced *dg* ncRNAs (S5 Fig, WT/anti-Myc). The amount of steady-state intron-containing *dg* ncRNAs was lower than that of intron-less *dg* ncRNA (Fig 5B, lanes WT). There was no difference in the amount of intron-less *dg* ncRNA between the WT and *Δdcr1*, whereas the amount of intron-containing *dg* ncRNA was significantly elevated in *Δdcr1* (Fig 5B, lanes *Δdcr1*). If the amounts of both transcripts were the same, these results imply that *dg* transcripts from the intron-containing minichromosome were more efficiently processed into siRNAs than those from the intron-less minichromosome. In addition, the decreased binding of spPrp16p to *dg* ncRNA was observed in *Δdcr1* (Fig 5A), suggesting that the interaction between spPrp16p and *dg* ncRNA was dependent on a functional RNAi system.

Previous studies reported that the formation of centromeric heterochromatin is important for preventing the missegregation of chromosomes during mitosis and meiosis [12, 13]. To evaluate the role of the *dg* intron in the formation of centromeric heterochromatin, we performed mitotic stability assays on minichromosomes. Specifically, we measured the frequency of mitotic losses in the minichromosome with or without the *dg* intron. Because the minichromosome used in these experiments contained the *leu1* selection marker, mitotic loss of the minichromosome could be analyzed by growing minichromosome-containing cells on plates with or without leucine. The results obtained indicated that the frequency of missegregation of the intron-less minichromosome (Less) was higher than that of the intron-containing minichromosome (Full) in wild-type cells (Fig 5C). We also observed the same phenomenon in *Δdcr1* cells, suggesting that the effect of the *dg* intron on chromosome segregation is at least partially RNAi-independent. Collectively, these results led us to conclude that the intron in *dg* ncRNA facilitates formation of centromeric heterochromatin in *S*. *pombe*.

### Analysis of a chimeric RNA containing the intron of the euchromatic gene

**Fig 6:**
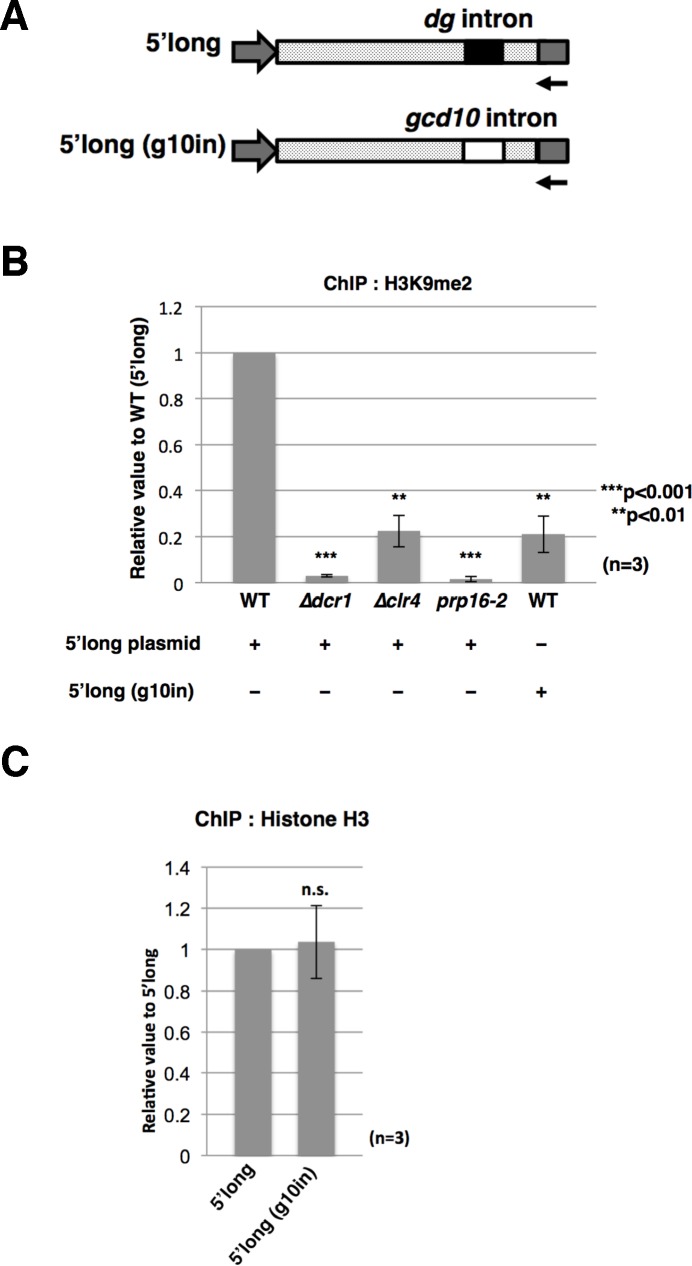
The intron sequence is important for facilitated H3K9 dimethylation. (A) Schematic structures of the 5’-long construct that expresses SPNCRNA.232 with the 570 bp upstream sequence, *dg* intron, and 106 bp downstream sequence and the 5’-long (g10in) construct containing the *gcd10* intron replaced with the *dg* intron. The arrow indicates the position of the plasmid-specific reverse primer. (B) ChIP analysis of H3K9me2 on the plasmids. The level of H3K9me2 in the 5’-long construct was reduced in the RNAi mutants (*Δdcr1* and *Δclr4*) and *prp16-2*. The 5’-long (g10in) construct had a reduced level of H3K9me2 in wild-type cells. (C) ChIP analysis of histone H3 on the 5’-long and 5’-long (g10in) constructs. qRT-PCR was carried out using plasmid-specific primers.

To examine the role of the *dg* intron in facilitating H3K9 dimethylation, we replaced the *dg* intron in the *dg* ncRNA gene (SPNCRNA.232) cloned in pREP1 (the 5’-long construct) with the intron from the *gcd10* gene, which encodes a tRNA methyltransferase (Fig 6A). We selected the *gcd10* intron because the intron length (136 bp) is similar to that of the *dg* intron (138 bp). As shown in S6 Fig, the chimeric transcript from the fusion construct, 5’-long (g10in), was spliced with low efficiency, although the *gcd10* intron itself is spliced efficiently in the original *gcd10* pre-mRNA. ChIP analysis using plasmid-specific primers revealed that a H3K9me2 level on the 5’-long construct was significantly reduced in *Δdcr1*, *Δclr4*, and *prp16-2*, suggesting that enhanced methylation of H3K9 on the plasmid construct is RNAi- and spPrp16p-dependent, like the minichromosome constructs (Fig 6B). Interestingly, the 5’-long (g10in) construct also exhibited a reduced level of H3K9me2 relative to that of the 5’-long construct [Fig 6B, WT/5’-long and WT/5’-long (g10in)]. By contrast, histone H3 on the 5’-long (g10in) construct was almost the same as that on the 5’-long construct (Fig 6C). These results suggest that the sequence of the *dg* intron itself is important for H3K9 dimethylation.

To identify the sequence element involved in the facilitated H3K9 dimethylation, we replaced a part of the *dg* intron in the 5’-long construct with the sequence of the *gcd10* intron, as shown in S7A Fig, and performed ChIP analysis using the plasmid-specific primers. Replacement of the 5’ side sequence (46 bp) of the *dg* intron with that of the *gcd10* intron decreased the level of H3K9me2, suggesting that an element responsible for facilitating H3K9 dimethylation is present in the 5’ region of the intron (S7B Fig).

### Mutations at splice sites affect the efficiency of H3K9 methylation

**Fig 7:**
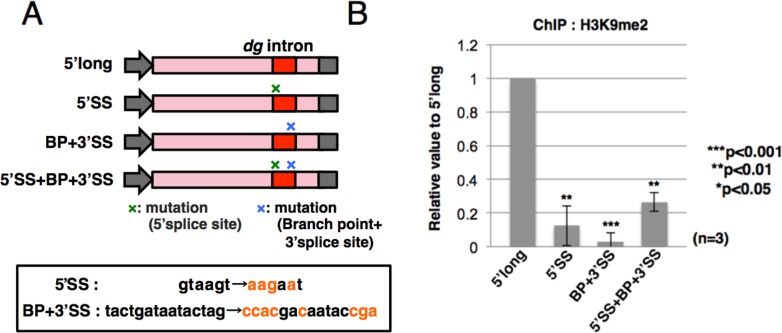
Mutations at the splice and branch sites decrease the efficiency of H3K9 dimethylation. (A) Schematic representation of mutation sites in the 5’-long construct. Mutated nucleotide sequences of the 5’ splice (5’SS), branch (BP), and 3’ splice (3’SS) sites are shown below the schematic. (B) ChIP analysis of H3K9me2 at the plasmids was performed using strains harboring plasmids with the indicated mutations. qPCR was performed using specific primers.

To determine whether recognition of splice sites in the *dg* intron is required to facilitate heterochromatin formation, we constructed plasmids containing the *dg* intron with mutated splice sites. As shown in Fig 7A, we mutated the 5’ splice site (5’SS), branch and 3’ splice sites (BP+3’SS), or all of them (5’SS+BP+3’SS) in the *dg* intron. All of these mutations completely inhibited splicing of the *dg* transcripts (S8A Fig). Interestingly, ChIP analysis revealed that the level of H3K9me2 at the *dg* region was significantly reduced in plasmids containing mutations at the splice and branch sites, in comparison with plasmid containing the wild-type *dg* intron (Fig 7B). In addition, RIP analysis using a strain expressing Myc-tagged spPrp16p revealed that association of spPrp16p with the *dg* ncRNA was significantly decreased by the mutations at the 5’SS, BP+3’SS and 5’SS+BP+3’SS (S8B Fig). These results suggest that the splice and branch site sequences recognizable by splicing factors are necessary for the facilitation of H3K9 dimethylation, in addition to the 5’ *cis*-element in the *dg* intron.

### An upstream region is involved in the splicing repression of *dg* ncRNA

In *S*. *pombe*, more than 43% of genes contain introns [14]. However, in contrast to the *dg* intron, the introns in these euchromatic genes do not induce the formation of heterochromatin. What is the difference between the intron in the *dg* ncRNA gene and those in euchromatic genes? In this regard, we noted that the splicing efficiency of *dg* ncRNA is very low (S5 Fig, lane: Input; S6 Fig, lane: 5’-long; [6]), despite the fact that the splice and branch site sequences in the *dg* intron closely match the corresponding consensus sequences in *S*. *pombe* [15]. This low splicing efficiency appears to be a unique feature of the *dg* intron, as most introns in the euchromatic regions are efficiently spliced after transcription. Interestingly, we found that truncated *dg* ncRNA transcribed from a plasmid with short upstream and short downstream sequences driven by the *nmt1* promoter was efficiently spliced, similar to euchromatic introns (S9A and S9B Fig, *dg*-short). This efficient splicing was not due to the *nmt1* promoter, because original-size *dg* ncRNA transcribed under the control of the *nmt1* promoter exhibited very low splicing efficiency, similar to endogenous *dg* ncRNA (S9A and S9B Fig, *dg* long). The plasmid producing efficiently spliced *dg* ncRNA exhibited a markedly reduced level of H3K9me2 in the ChIP analysis (S9C Fig, Short) relative to the plasmid possessing the long construct with low splicing efficiency (S9C Fig, Long), suggesting that retention of the *dg* intron by splicing repression facilitates dimethylation of H3K9.

**Fig 8:**
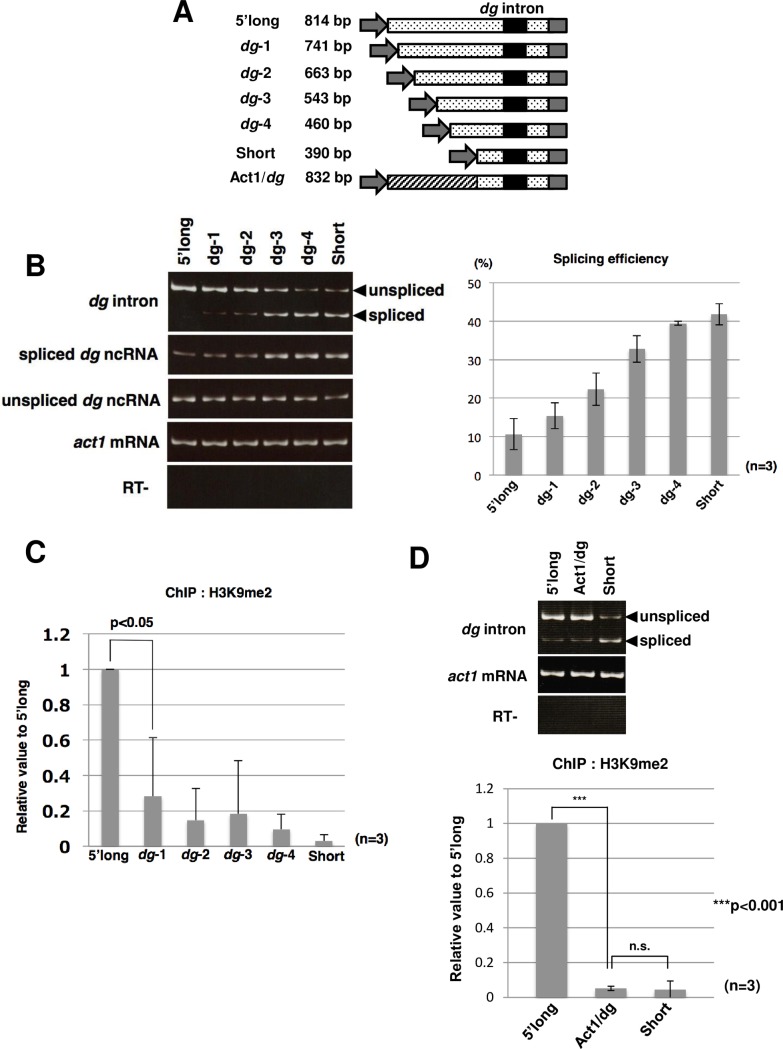
Deletion of the upstream region affects pre-mRNA splicing and H3K9 dimethylation. (A) Schematic representation of *dg* fragments containing serial deletions in the upstream region. Constructs were fused to the *nmt1* promoter and terminator (gray arrows and boxes, respectively). Numbers on the left represent the lengths of intron-containing *dg* DNA fragments and the Act1/*dg* chimeric fragment ligated to the *nmt1* promoter and terminator. The 5’-long construct consists of the upstream region (570 bp), *dg* intron (138 bp), and downstream region (106 bp). The Act1/*dg* construct consists of the actin gene fragment (442 bp) and the *dg*-short fragment (390 bp). (B) Splicing of *dg* ncRNAs transcribed from serially deleted fragments was examined by RT-PCR (left panel) and qRT-PCR (right panel) using plasmid-specific primers. Products specific for spliced *dg* ncRNA were amplified using spliced product-specific primers. The ratios of the spliced products to the total transcripts (spliced and unspliced products) were calculated and graphed. *Act1* mRNA was analyzed as a loading control. No bands were detected in samples without the reverse transcription reaction. (C) ChIP analysis of H3K9me2 at the *dg* region was performed with the H3K9me2 antibody and strains possessing the indicated plasmids. qPCR was performed using plasmid-specific primers. (D) Splicing of the Act1/*dg* chimeric transcript was analyzed by RT-PCR (upper panel) and qRT-PCR (lower panel). Splicing of 5’-long and *dg*-short transcripts were also examined as controls.

Because a 5’-long construct with the long upstream sequence (-570 bp from the *dg* intron) and shortened downstream sequence (+106 bp from the *dg* intron) exhibited low splicing efficiency, similar to the wild-type *dg* construct (*dg*-long), we speculated that the upstream region contains the *cis*-element needed for splicing repression to retain the intron element facilitating H3K9me2. Because splicing repression of the 5’-long construct was not relieved in *Δdcr1* and *Δclr4*, inhibition of *dg* RNA splicing is not dependent on the RNAi system (S10 Fig). To identify the *cis*-elements responsible for splicing repression, we constructed a series of plasmids expressing *dg* transcripts with serially deleted upstream regions (Fig 8A). The transformants with these plasmids were then subjected to RT-PCR, qRT-PCR and ChIP analyses. As a result, we found that deletion of the upstream region gradually increased splicing efficiency (Fig 8B) and decreased the H3K9me2 levels (Fig 8C), implying a tendency toward an inverse relationship between the splicing efficiency of the *dg* intron and H3K9 modification.

We also constructed a deletion series using the 5’-long (g10in) construct with the *gcd10* intron and examined their splicing efficiencies. As shown in S11 Fig, deletions of the upstream region promoted splicing efficiency of the *gcd10* chimeric constructs, although splicing enhancements in *dg-4* and *short* chimeric transcripts were weak for unknown reasons. This result suggests that splicing repression is independent of intron sequences.

The gradual increase of splicing efficiency in the 5’-long deletion constructs suggests that the upstream region contains no specific *cis*-elements involved in repression, and that the distances between the transcription start sites and the intron are important for the splicing repression. To investigate this possibility, we ligated a 442 bp DNA fragment of the actin gene (+4 to +445) upstream of *dg*-short to yield the Act1/*dg* construct, which has almost the same upstream length as 5’-long but with a different sequence (Fig 8A). RT-PCR analysis revealed that the splicing efficiency of Act1/*dg* transcripts was very low, like the 5’-long transcripts (Fig 8D), indicating that distance from the transcription start site to the *dg* intron is important for splicing repression. Moreover, ChIP analysis revealed that the level of H3K9 dimethylation on the Act1/dg construct was low (Fig 8D), suggesting that elements involved in promoting formation of H3K9me2 are present not only in the *dg* intron but also in the 5' upstream region.

### Intron-containing centromeric ncRNAs are inefficiently spliced

**Fig 9:**
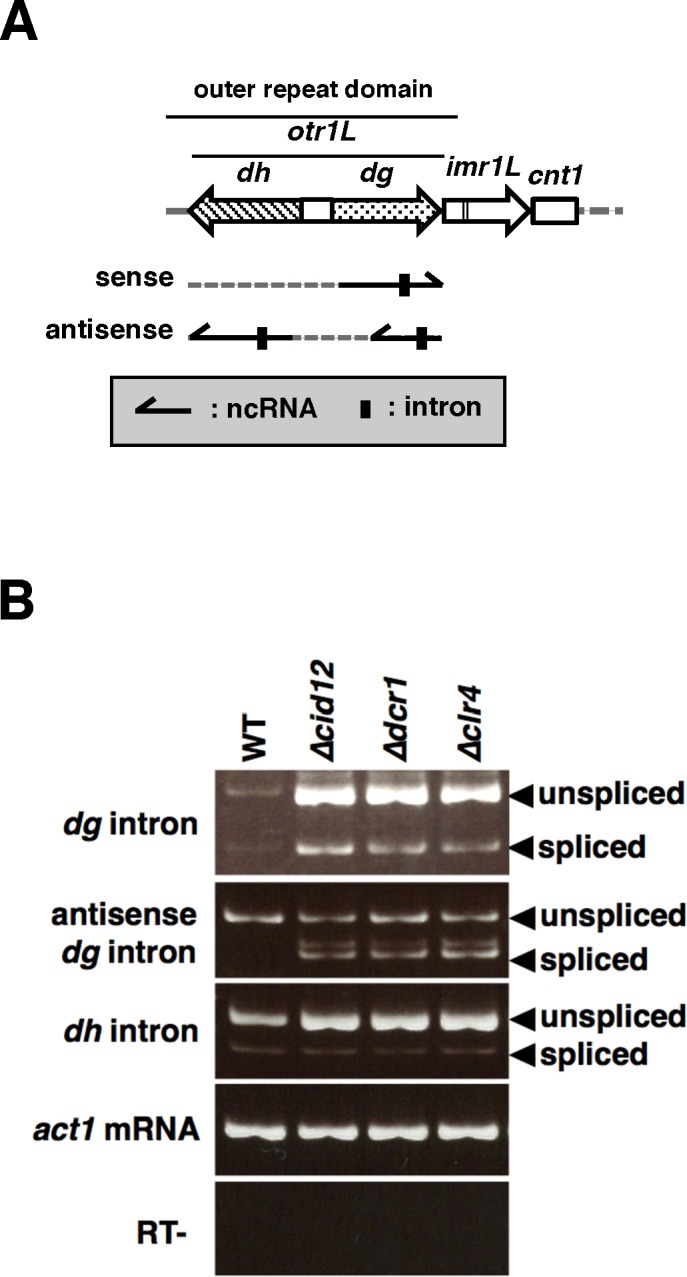
Inefficient splicing of centromeric introns. (A) Schematic representation of introns in centromeric ncRNAs. Arrows indicate the direction of transcription. Vertical boxes denote the intron. (B) RT-PCR analyses of the *dg* and *dh* transcripts and *dg* antisense transcript containing the intron. Splicing efficiencies of the transcripts are low in wild-type cells. Deletion of the *cid12*^*+*^, *dcr1*^*+*^, and *clr4*^*+*^ genes, which are essential for RNAi-mediated formation of heterochromatin, promoted splicing of the *dg* antisense transcript. *Act1* mRNA was detected as a loading control (*act1* mRNA). “RT-” represents the reaction without reverse transcriptase.

Recently, Lee *et al*. showed that “cryptic introns” specify heterochromatin domains (HOODs) in the *S*. *pombe* genome [16]. In particular, they reported the presence of the “cryptic introns” in the *dh* transcript and *dg* antisense transcript [16] (Fig 9A). We examined splicing of these intron-containing centromeric transcripts by RT-PCR and found that their splicing efficiencies were also low, like the *dg* intron, in wild-type cells (Fig 9B, WT). Interestingly, in a strain harboring deletion of the *cid12*^*+*^, *dcr1*^*+*^, or *clr4*^*+*^ genes, which are essential for the RNAi machinery, splicing of the antisense *dg* intron was significantly upregulated (Fig 9B, *Δcid12*, *Δdcr1*, and *Δclr4*), suggesting that its splicing is also repressed in wild-type cells, like the *dg* intron, and that the RNAi system is involved in that splicing repression. Overexpression of Dcr1p and Cid12p, by contrast, did not affect splicing of centromeric ncRNAs in wild-type cells (S12 Fig).

## Discussion

In this study, we cloned the *S*. *pombe* gene responsible for the *prp14* mutation, which impairs RNAi-mediated formation of centromeric heterochromatin (Figs 1 and 2), as well as pre-mRNA splicing [8]. We found that the *prp14*^+^ gene encodes a homologue of the *S*. *cerevisiae* and human splicing factor Prp16p, a DEAH-box RNA helicase with ATPase activity [17] (S1C Fig). We, therefore, renamed *prp14* as *prp16*. Although *prp16* exhibited defective splicing of pre-mRNAs encoding several factors involved in RNAi-mediated gene silencing (S3 Fig), the observation that spPrp16p (encoded by the *prp16*^+^ gene) is enriched at the centromere and interacts with Cid12p, a component of the RDRC of the RNAi system, suggested that spPrp16p plays a direct role in the formation of centromeric heterochromatin (Fig 3). Thus, defective formation of centromeric heterochromatin in *prp16* might be caused by composite effects of the splicing impairments and the functional abnormality of spPrp16p in heterochromatin formation.

We previously reported that another temperature-sensitive splicing mutant, *prp13*-*1*, in which the affected gene encodes the U4 snRNA essential for pre-mRNA splicing [18], is defective in centromeric gene silencing [6]. In the case of *prp13*, splicing impairments for RNAi factors were not observed at the temperature (26°C) that induced defective formation of centromeric heterochromatin (S3 Fig). Therefore, the phenotype defective in the formation of centromeric heterochromatin in *prp13-1* does not appear to be a secondary effect of splicing defects, although we cannot exclude the possibility that pre-mRNA splicing of unknown factors involved in gene silencing is defective in *prp13-1*.

Several studies have reported crosstalk between splicing factors and RNAi- or exosome-mediated gene silencing. The first such evidence came from pull-down experiments using TAP-tagged Cid12p, which showed that several spliceosomal proteins co-precipitated with Cid12p-TAP in *Δrdp1* [10]. Bayne *et al*. [5] revealed that *csp4* (centromere: suppressor of position effect 4) and *csp5*, which alleviated the silencing of marker genes inserted into the *otr* region in the centromere, encode the splicing factors Cwf10p and Prp39p, respectively. They also demonstrated that Cwf10p associates with Cid12p and centromeric chromatin, suggesting that splicing factors facilitate RNAi-directed silencing [5]. Another splicing factor, Spf30p, was also shown to assist exosome-mediated centromeric gene silencing [19]. In addition, in the yeast *Cryptococcus neoformans*, spliceosomes stalled on intron-containing pre-mRNAs serve as a signal that induces siRNA synthesis by the SCANR (Spliceosome-Coupled And Nuclear RNAi) complex [20]. It remains unclear why these splicing factors associate closely with RNAi- or exosome-mediated gene silencing. In our previous study on *prp13-1*, we identified an intron typical for those in pre-mRNAs in centromeric *dg* ncRNA, and proposed a model in which the spliceosome or sub-spliceosome assembled on the intron functions as a platform for recruiting RDRC to facilitate the processing of centromeric ncRNAs [6]. In this study, we tested this platform model by removing the intron from the *dg* transcript and mutating the splice sites of the *dg* intron, leading to reduced binding of spPrp16p to *dg* ncRNA (Fig 5A and S8B Fig). The results showed that the removal of and mutations in the intron significantly decreased levels of H3K9me2 and Swi6p binding (Figs 4B and 7B), indicating that the intron is actually necessary for facilitating RNAi-mediated formation of heterochromatin.

Lee *et al*. recently suggested that the splicing machinery and its associated factor Nrl1p (NRDE-2 like 1) act on introns to specify domains for heterochromatin (HOODs) in *S*. *pombe* [16]. They discovered widespread previously unannotated “cryptic introns” in genes for mRNAs and ncRNAs, including the *dh* ncRNA gene in the pericentromere [16]. The *dh* ncRNAs, as well as *dg* ncRNAs, are processed to siRNAs essential for RNAi-mediated heterochromatin formation. They proposed that RNAi targets include “cryptic introns”, which play an important role in defining the targets of RNAi/exosome-mediated heterochromatin assembly through the spliceosome [16]. Our results and the aforementioned findings support the idea that centromeric introns serve as signals for heterochromatin formation, in collaboration with the splicing machinery.

We found that splicing of the *dg* ncRNA was affected by the distance from the transcription start site to the intron (Fig 8). The chimeric 5’-long (g10in) and Act1/*dg* constructs also exhibited low splicing efficiencies, supporting the idea that splicing repression of the *dg* intron depends on the length, rather than the nucleotide sequence, of the upstream sequence (S6 and S11 Figs and Fig 8D). Several lines of experiments in yeast and mammals demonstrated that a cap-binding complex (CBC) promotes binding of U1 snRNP to cap-proximal 5' splice sites and stimulates splicing; consequently, introns positioned closely to transcription start sites are spliced more efficiently [21, 22]. Consistent with this, in single-intron genes in yeasts, most introns are found at the 5' ends [23]. Thus, the downstream positioning of the *dg* intron may keep splicing efficiency low so that the intron is retained within the transcript.

qRT-PCR and ChIP analyses of the 5’ deletion constructs showed that splicing efficiency of the *dg* intron and H3K9me2 levels tended to have an inverse relationship (Fig 8B and 8C). Deletion of the upstream region increased splicing efficiency gradually; however, the H3K9me2 levels decreased abruptly after deletion of 73 bp from the 5’long construct, suggesting that there exists a threshold splicing efficiency for the enhancement of heterochromatin formation or that a *cis*-element facilitating heterochromatin formation, which functions in combination with the *dg* intron, is present in the 5’ upstream region.

**Fig 10:**
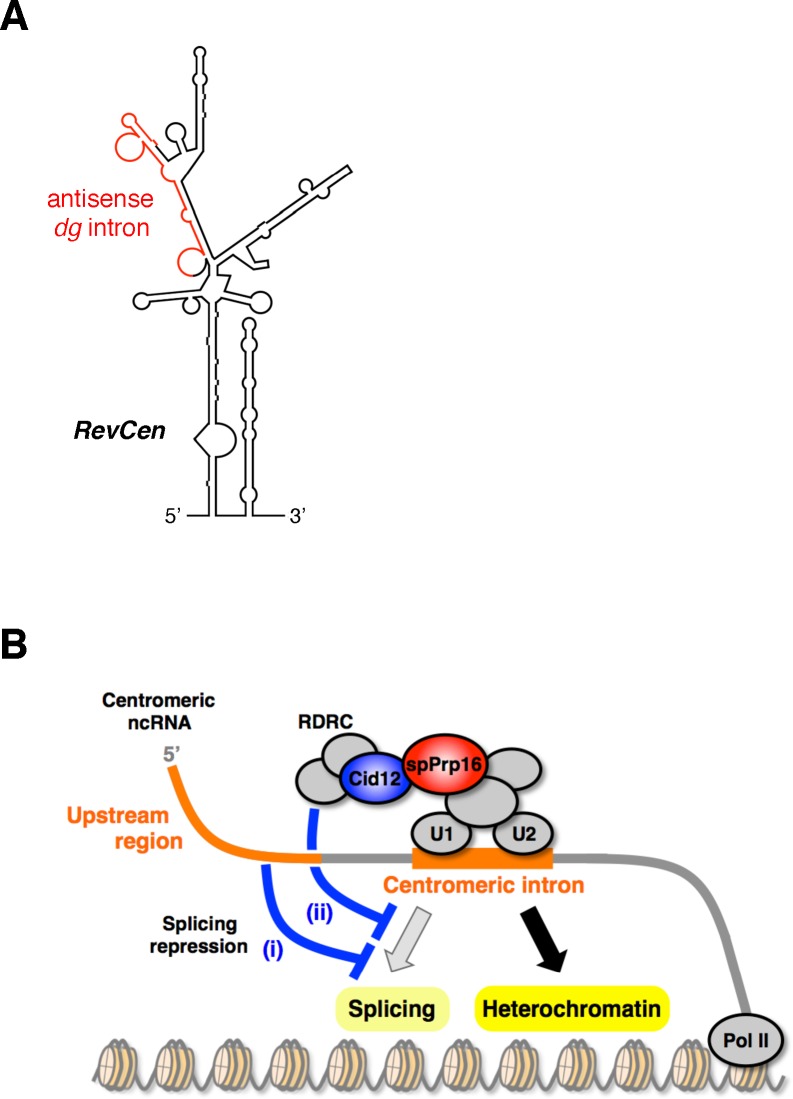
Model showing how the intron in centromeric ncRNAs facilitates RNAi-mediated formation of heterochromatin. (A) Predicted hairpin-like structures of *RevCen* [24] containing the antisense *dg* intron. Secondary structure of *RevCen* predicted by Mfold [25] was assessed experimentally [24]. Intron regions are indicated in red. (B) Efficient recruitment of RNAi components onto centromeric ncRNA is achieved through their interaction with the spliceosome (or sub-spliceosome) assembled on the intron. Repression of the splicing reaction is induced by the long upstream region (i) or induced by the active RNAi machinery, resulting in formation of a hairpin-like structure in the intron region, which might be processed into siRNAs RDRC-independently as proposed in [24]. Then, the stalled splicing complex may promote the association of RNAi components with ncRNAs, facilitating heterochromatin formation.

We found that the “cryptic introns” also had very low splicing efficiencies (Fig 9). It is noteworthy that depletion of a factor essential for the RNAi machinery, such as Cid12p, Dcr1p, or Clr4p, facilitated splicing of the antisense *dg* transcript (Fig 9B). This result suggested that the active RNAi machinery is closely involved in splicing repression for the antisense *dg* intron. Interestingly, the antisense *dg* intron is localized within the hairpin-like, highly structured region called *RevCen* (Fig 10A) [24]. Small centromeric RNAs derived from the *RevCen* region were found in *Δrdp1* cells, suggesting that hairpin-like *RevCen* transcripts are processed into siRNAs by Dcr1p, independently of RDRC activity [24]. These siRNAs have been proposed to function in initiation of heterochromatin formation [24]. It is possible that removal of the antisense *dg* intron from *RevCen* transcripts by splicing disrupts the hairpin-like structure necessary for RDRC-independent processing, resulting in reduced formation of heterochromatin.

Furthermore, regions containing the *dg* and *dh* introns were also predicted by Mfold, which predicts RNA folding by minimizing free energy [25], to form highly structured hairpins (S13 and S14 Figs), although the predicted secondary structures should be confirmed experimentally, as was done previously for *RevCen* structure using enzymatic and chemical probing [24]. The centromeric introns retained in the transcripts by splicing repression may function not only in the splicing machinery-dependent facilitation of heterochromatin formation via assembly of the platform complex, but also in RDRC-independent initiation of heterochromatin assembly through formation of hairpin-like RNAs that are processed directly by Dcr1p as proposed for *RevCen* [24].

Splicing repression may result in formation of a stalled spliceosome, triggering association and recruitment of RNAi factors to centromeric ncRNAs and promoting the formation of heterochromatin (Fig 10B). Given that the 5’-long (g10in) and Act1/*dg* transcripts did not induce facilitated H3K9 dimethylation, the *cis*-elements in the *dg* intron and 5' upstream region seem to be required for upregulation of H3K9 dimethylation, in addition to repression of splicing (S7 Fig and Fig 8). Further analyses of the *cis*-elements and the mechanisms that connect splicing repression to formation of heterochromatin are now underway in our laboratory.

Interestingly, the human homologue of spPrp16p, DHX38, is a component of the Interphase Centromere Complex (ICEN) [26], suggesting that the human homologue of spPrp16p also plays an important role at the centromere. We recently found that satellite I ncRNAs transcribed from human centromeres associate with Aurora B kinase, a major regulator of chromosome segregation, and that knockdown of centromeric satellite I ncRNAs induces the defective attachment of microtubules to kinetochores, leading to impaired segregation of chromosomes in HeLa cells [27]. Association of DHX38 with satellite I ncRNAs is now under investigation. Analyses of spPrp16p, together with its human homologue DHX38, and introns in centromeric ncRNAs will shed light on evolutionarily conserved crosstalk between the splicing machinery and the mechanisms that maintain centromere functions through ncRNAs.

## Materials and methods

### *S*. *pombe* strains, plasmids, and general methods

The *S*. *pombe* strains and plasmids used in this study are listed in S1 and S2 Tables, respectively. The general methods used for analyses of *S*. *pombe* are described in refs. [28] and [29].

### TBZ sensitivity test and silencing assay

For the TBZ sensitivity test, cells were suspended in YE broth at a density of 2 × 10^3^ cells/ml, serially 5-fold diluted, and spotted on plates containing TBZ (10 μg/ml). In the silencing assay, serially diluted cells were spotted on N/S plates (non-selective YE medium supplemented with adenine, leucine, and uracil) or 5-FOA plates (N/S plates containing 1 mg/ml 5-FOA). The plates were incubated at 33°C for 3–4 days.

### RT-PCR analysis

Cells were cultured to mid-log phase in YE or MMAU medium at 26°C [for *972*(WT), *Δdcr1*, *Δclr4*, and *Δcid12*] or 33°C (for *prp16-2*). For the splicing analysis in S3 Fig, the indicated strains were cultured at 26°C (*prp16-2*, 30°C), and then shifted to 22°C or 30°C (*prp16-2*, 22°C or 26°C) for 6 hours. To isolate total RNA, cells were harvested by centrifugation and treated with phenol: chloroform (5:1). After treatment with DNase I (Ambion), reverse transcription (RT) was performed using 1 μg of total RNA and a cDNA synthesis kit (TaKaRa). Random primers were used for reactions with *act* mRNA and the mRNAs in S3 Fig. Specific primers were used for RT of *dg* and *dh* ncRNAs, transcripts from the minichromosomes, and plasmid constructs. RT samples were then subjected to PCR amplification using the specific primers listed in S3 Table.

Splicing efficiencies of *dg* ncRNAs with upstream deletions were quantitated by qRT-PCR of total and spliced *dg* ncRNAs. Primers corresponding to the *dg* second exon and downstream vector region (the *nmt1* terminator region) were used for amplification of total products, and a primer spanning the exon junction and a primer complementary to the downstream vector region were used for amplification of the spliced products. The 5’-long plasmid without the intron was used for the qPCR standard for total and spliced products. The ratio of spliced *dg* ncRNA to total *dg* ncRNA was calculated and graphed. The assay was performed three times independently.

### Chromatin Immunoprecipitation (ChIP) analysis

ChIP analysis of H3K9me2, Swi6p, and spPrp16p-Flag was performed as described [6]. Extracts were prepared from cells cultured at 33°C (*prp16-2*) or 26°C (*Δdcr1*, *Δclr4*, *Δago1*, and wild-type *972*). Antibodies against H3K9me2 (ab1220) and FLAG (F3165) were purchased from Abcam and Sigma, respectively. Anti-Swi6p antibody was produced by Dr. Jun-ichi Nakayama. Real-time quantitative PCR was carried out with a Roche Diagnostic LightCycler using the primers listed in S3 Table. Data analyses were performed using the LightCycler Software (ver. 3.5). ChIP analyses were conducted at least three times independently.

### RNA Immunoprecipitation (RIP) analysis

A minichromosome was introduced into cells expressing spPrp16-Myc from the endogenous locus. Cells (972 or *Δdcr1*) transformed with the indicated constructs were grown to mid-log phase in MMAU medium at 26°C, and then fixed with 1% formaldehyde for 30 min at room temperature. After treatment with 125 mM glycine, cells were washed with PBS and resuspended in lysis buffer (50 mM HEPES-KOH, pH 7.5, 140 mM NaCl, 1% TritonX-100, 0.1% DOC, 1 mM PMSF, complete EDTA-free protease inhibitor cocktail, and 100 U/ml RNase inhibitor). Cells were then disrupted using a Multi-beads shocker (Yasui Kikai), followed by sonication with a Bioruptor (BM Equipment). After treatment with DNase (Ambion), spPrp16-Myc was immunoprecipitated using anti c-Myc antibody (9E10, Santa Cruz), and RNAs were extracted from the precipitates. RT of RNAs was performed using the plasmid-specific primers listed in S3 Table. Real-time quantitative PCR and data analyses were performed as described above in the “ChIP analysis” section. RIP analyses were conducted at least three times independently.

### Immunostaining

Immunostaining of tubulin in the *prp16-2* mutant was performed as described previously [6]. The TAT1 monoclonal antibody against tubulin was provided by Dr. Keith Gull. After counterstaining with DAPI, cells were observed with an Olympus AX70 fluorescence microscope equipped with a Photometrics Quantix-cooled CCD camera.

### Mitotic stability assay

Cells containing the minichromosome with or without the *dg* intron were cultured to mid-log phase in MMAU medium at 26°C. Cells (300 cells/plate) were plated on MMAU and MMALU plates, and then incubated at 26°C for 10 days. Colonies grown on the plates were counted to calculate the mitotic stability of the minichromosome. The number of colonies on MMAU plates was divided by the number of colonies on MMALU plates.

## Supporting information

S1 Text Cloning of the *prp14^+^* gene. (DOCX)

S1 Fig Cloning of the *prp14^+^* gene. (A) Schematic structure of the genomic fragment in pSP#1. The subcloned regions and results of functional complementation are shown below the gene structure. The subcloned fragments that rescued defective growth at the non-permissive temperature are indicated as Ο, and non-rescuing fragments are indicated as X. (B) Complementation test of the subcloned fragments. The fragment containing the spPrp16p gene rescued the cold-sensitive phenotype. Prp16-pSP1 indicates the *prp16* mutant transformed with pSP1 vector. (C) Amino acid alignment of *S*. *pombe* spPrp16p, *S*. *cerevisiae* Prp16p, and human DHX38/Prp16p. DEAH-box helicase and helicase superfamily C-terminal domains are underlined. Identical and similar amino acids conserved among species are colored in red. Amino acids conserved between two species are shown in blue.
(PDF)

S2 Fig A schematic of the centromere region with the site of the *ura4^+^* insertion used for the silencing assay. The *ura4*^*+*^ gene was inserted into the *otr1R* region of chromosome 1.
(PDF)

S3 Fig Analysis of defective splicing of pre-mRNAs encoding factors involved in RNAi-induced formation of centromeric heterochromatin. Total RNAs were isolated from the indicated strains cultured at 22°C, 26°C, or 30°C and then subjected to RT-PCR analysis using primers specific for the indicated intron regions.
(PDF)

S4 Fig Localization of spPrp16p-GFP expressed from the endogenous locus, co-immunoprecipitation and RNase treatment. (A) spPrp16p-GFP expressed from the endogenous locus localized diffusely in the nucleus. No dot-like signals were observed. Cells cultured at 30°C were counterstained with Hoechst 33342 and observed through a Nikon Eclipse Ti fluorescence microscope equipped with an ORCA-R2 cooled CCD camera. (B) spPrp16-HA does not associate with Chp1p-FLAG. Co-immunoprecipitation analysis was carried out using a strain expressing spPrp16-HA and Chp1p-FLAG. spPrp16-HA was immunoprecipitated with (IP: anti-HA) or without (IP: -) an anti-HA antibody. The precipitates were then subjected to western blot analysis using anti-FLAG antibody. No band was detected in the precipitates with the anti-HA antibody, suggesting no association between spPrp16p and Chp1p. Arrowheads indicate positions of spPrp16-HA and Chp1p-FLAG. (C) Treatment of extracts with RNase A degraded *dg* ncRNA. Extracts from cells with (spPrp16-HA: +) or without (spPrp16-HA: -) a plasmid expressing spPrp16-HA were treated with 10 μg/ml RNase A at 37°C for 10 min (RNase A: +), and then subjected to the RT-PCR analysis. “RT-” indicates a reaction without reverse transcriptase.
(PDF)

S5 Fig spPrp16p binds unspliced and spliced *dg* ncRNA. RT-PCR products prepared from the indicated precipitates were electrophoresed on an 8% polyacrylamide gel and stained with ethidium bromide. RNA immunoprecipitation was performed using anti-Myc antibody. “RT-” indicates a reaction without reverse transcriptase.
(PDF)

S6 Fig Splicing of the transcripts from the 5’-long and 5’-long (g10in), which contains the *gcd10* intron, is not efficient. RT-PCR was performed using plasmid-specific primers. *Act1* mRNA was detected as a loading control (*act1* mRNA). “RT-” indicates a reaction without reverse transcriptase.
(PDF)

S7 Fig The 5’ region of the *dg* intron is important for facilitation of H3K9 dimethylation. (A) Schematic representation of the chimeric 5’-long constructs, in which part of the intron was replaced with the sequence of the *gcd10* intron (white boxes). (B) ChIP analysis of H3K9me2 on the chimeric 5’-long constructs. The 5’-long (g10in-A) construct in which the 5’ region of the *dg* intron was replaced with the corresponding region of the *gcd10* intron had a reduced level of H3K9me2.
(PDF)

S8 Fig Analysis of the *dg* ncRNAs with mutated splice sites. (A) Splicing of the *dg* ncRNA was completely inhibited by the mutations introduced. RT was performed using plasmid-specific primers. PCR was conducted using primers that amplified the region spanning the intron (upper panel), which were used for evaluation of the splicing reaction, or primers that amplified the region downstream of the intron (middle panel), which were used to determine the amounts of the transcripts. *act1* mRNA was analyzed as a loading control (bottom panel). “RT-” indicates a reaction without reverse transcriptase. (B) RIP analysis of spPrp16-Myc was performed with the anti-Myc antibody (anti-Myc) or non-immune IgG (normal IgG) and strains possessing plasmids with the indicated mutations. qPCR was performed using plasmid-specific primers.
(PDF)

S9 Fig A *dg* transcript with the short flanking sequences exhibited more efficient splicing. (A) Schematic representation of plasmids expressing *dg* transcripts. The *dg*-long and *dg*-short constructs contain the 1,230 and 390 bp fragments including the *dg* intron, in addition to the *nmt1* promoter and terminator (gray arrow and box, respectively). (B) Splicing of *dg* ncRNAs expressed from plasmids or the endogenous locus was analyzed by RT-PCR using plasmid-specific primers (*dg*-long or *dg*-short) or endogenous gene-specific primers (endo. *dg*). The arrowheads indicate unspliced and spliced products. The *dg*-short transcript was spliced efficiently. No bands were detected in any samples without the reverse transcription reaction. *act1* was detected as a loading control. (C) ChIP analysis of H3K9me2 at the *dg* region was performed using the H3K9me2 antibody and strains harboring the *dg*-long or *dg*-short plasmid. qPCR was performed using plasmid-specific primers.
(PDF)

S10 Fig Inefficient splicing of the *dg* intron in the 5’-long construct in the RNAi-defective deletion mutants. Splicing of the 5’-long *dg* intron in the indicated strains was analyzed by RT-PCR. *Act1* mRNA was detected as a loading control (*act1* mRNA). “RT-” indicates a reaction without reverse transcriptase.
(PDF)

S11 Fig Deletion of the upstream region affects the splicing efficiency of the *gcd10* chimeric transcript. (A) Schematic representation of chimeric fragments containing the *gcd10* intron and serially deleted upstream region. Constructs were fused to the *nmt1* promoter and terminator (gray arrows and boxes). The numbers on the left denote the length of each intron-containing *dg* DNA fragment inserted between the *nmt1* promoter and terminator. (B) Splicing of the *gcd10* chimeric RNAs transcribed from serially deleted fragments was examined by RT-PCR (upper panel) and qRT-PCR (lower panel) using plasmid-specific primers. To draw a graph of splicing efficiency, the ratios of the spliced products to the total transcripts were calculated. In RT-PCR, *Act1* mRNA was analyzed as a loading control. “RT-” indicates a reaction without reverse transcriptase.
(PDF)

S12 Fig Overexpression of RNAi factors does not affect splicing of intron-containing centromeric ncRNAs. A multicopy plasmid (pSP1) harboring the *dcr1*^+^ or *cid12*^+^ gene was introduced into wild-type cells, and splicing of *dg*, antisense *dg* and *dh* ncRNAs was analyzed by RT-PCR (panels of *dg* intron, antisense *dg* intron and *dh* intron). Overexpression of the *dcr1*^+^ and *cid12*^+^ genes was confirmed by RT-PCR (panels of *dcr1* mRNA and *cid12* mRNA). *Act1* mRNA was analyzed as a loading control (*act1* mRNA). “RT-” indicates a reaction without reverse transcriptase.
(PDF)

S13 Fig Predicted structure of the *dg* intron region. Secondary structure of the *dg* intron with upstream (-100 bp from the *dg* intron) and downstream (+100 bp from the *dg* intron) sequences was predicted using Mfold (http://unafold.rna.albany.edu/?q=mfold/RNA-Folding-Form). Mutations introduced in the splice sites or branch point shown in Fig 7 did not affect the predicted structure significantly. Because this region is included in the *dg*-short construct, deletions of the upstream sequence shown in Fig 8A also do not affect the structural prediction. The intron region is indicated in red.
(PDF)

S14 Fig Predicted structure of the *dh* intron region. Secondary structure of the *dh* intron with upstream (-50 bp from the *dh* intron) and downstream (+200 bp from the *dh* intron) sequences was predicted using Mfold (http://unafold.rna.albany.edu/?q=mfold/RNA-Folding-Form). The intron region is indicated in red.
(PDF)

S1 Table Strains used in this study. (PDF)

S2 Table Plasmids used in this study. (PDF)

S3 Table Primers used in this study. (PDF)
