# Supplementary material for: The intron in centromeric noncoding RNA facilitates RNAi-mediated formation of heterochromatin
Source: PLoS Genet. 2017 Feb 23;13(2):e1006606. doi: 10.1371/journal.pgen.1006606 (PMC5322907; doi:10.1371/journal.pgen.1006606)
Supplement: S1 Text — (DOCX) [file pgen.1006606.s001.docx]

**S1 text**

**Cloning of the *prp14^+^* gene**

To isolate the gene responsible for the *prp14* mutation, we transformed the *prp14-2* mutant with a *S. pombe* wild-type genomic DNA library constructed in pSP1, and then identified three clones (#1 to #3) that grew well at the non-permissive temperature of 22°C. Sequence analysis of the plasmid recovered from transformant #1 revealed that the plasmid contained three genes, SPBC1711.17, SPBC17G9.02c, and SPBC17G9.03c, which encode an ATP-dependent RNA helicase homologous to *Saccharomyces cerevisiae* and human Prp16p, the RNA polymerase II accessory factor Cdc73p, and the cytoplasmic lysine-tRNA ligase Krs1p, respectively (S1A Fig). Transformation of subcloned DNA fragments containing each gene showed that the 3.9 kb DNA fragment containing SPBC1711.17, encoding the Prp16 homologue, complemented the cold-sensitive growth phenotype of the *prp14* mutant (S1B and C Fig).

To determine the mutation site, we amplified the entire region of SPBC1711.17 by PCR and directly sequenced the amplified products using several primers complementary to the coding sequence of the *prp14*^+^ gene. We found that C at the position +418 in the *prp14*^+^ gene in the *prp14-2* mutant was replaced with T, resulting in the conversion of a glutamine codon (CAA) to a nonsense codon (TAA). Identification of the mutation site in SPBC1711.17 indicated that it is not a multicopy suppressor for the mutation, but an authentic *prp14*^+^ gene that was renamed as *prp16*^+^.
