# Supplementary figures and images for: The intron in centromeric noncoding RNA facilitates RNAi-mediated formation of heterochromatin
Source: PLoS Genet. 2017 Feb 23;13(2):e1006606. doi: 10.1371/journal.pgen.1006606 (PMC5322907; doi:10.1371/journal.pgen.1006606)

S1 Figure

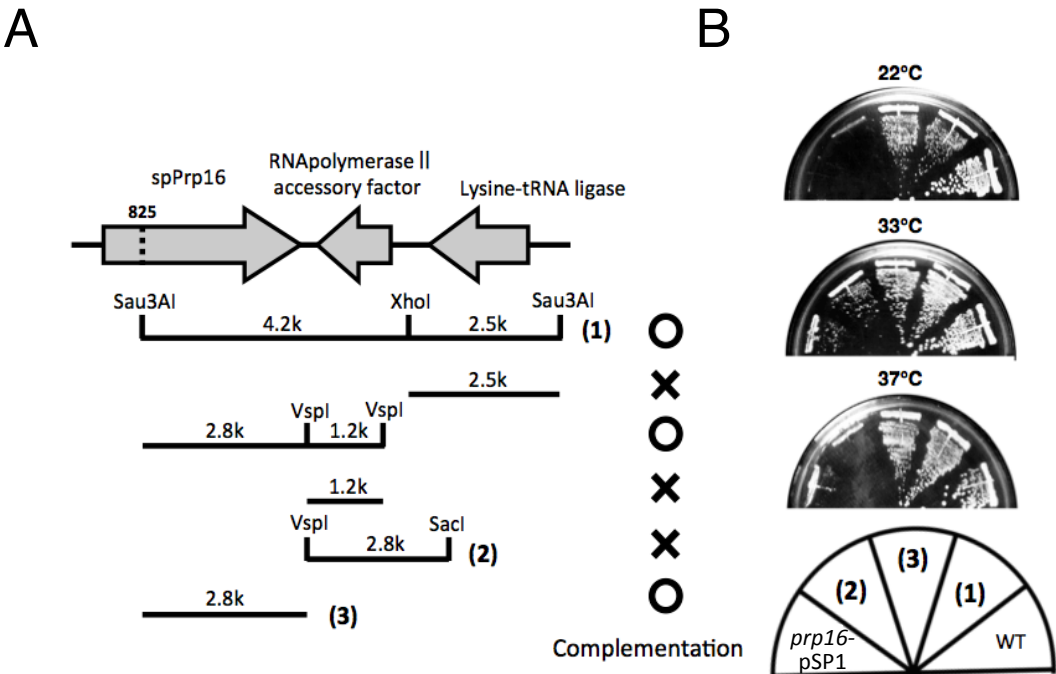

DEAH-box helicase

Helicase superfamily  
C-terminal

Supplement: S1 Fig — (A) Schematic structure of the genomic fragment in pSP#1. The subcloned regions and results of functional complementation are shown below the gene structure. The subcloned fragments that rescued defective growth at the non-permissive temperature are indicated as Ο, and non-rescuing fragments are indicated as X. (B) Complementation test of the subcloned fragments. The fragment containing the spPrp16p gene rescued the cold-sensitive phenotype. Prp16-pSP1 indicates the prp16 mutant transformed with pSP1 vector. (C) Amino acid alignment of S. pombe spPrp16p, S. cerevisiae Prp16p, and human DHX38/Prp16p. DEAH-box helicase and helicase superfamily C-terminal domains are underlined. Identical and similar amino acids conserved among species are colored in red. Amino acids conserved between two species are shown in blue. (PDF) [file pgen.1006606.s002.pdf]

# S2 Figure

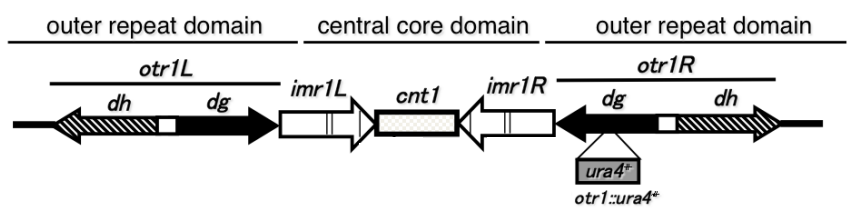

Supplement: S2 Fig — The ura4+ gene was inserted into the otr1R region of chromosome 1. (PDF) [file pgen.1006606.s003.pdf]

S3 Figure

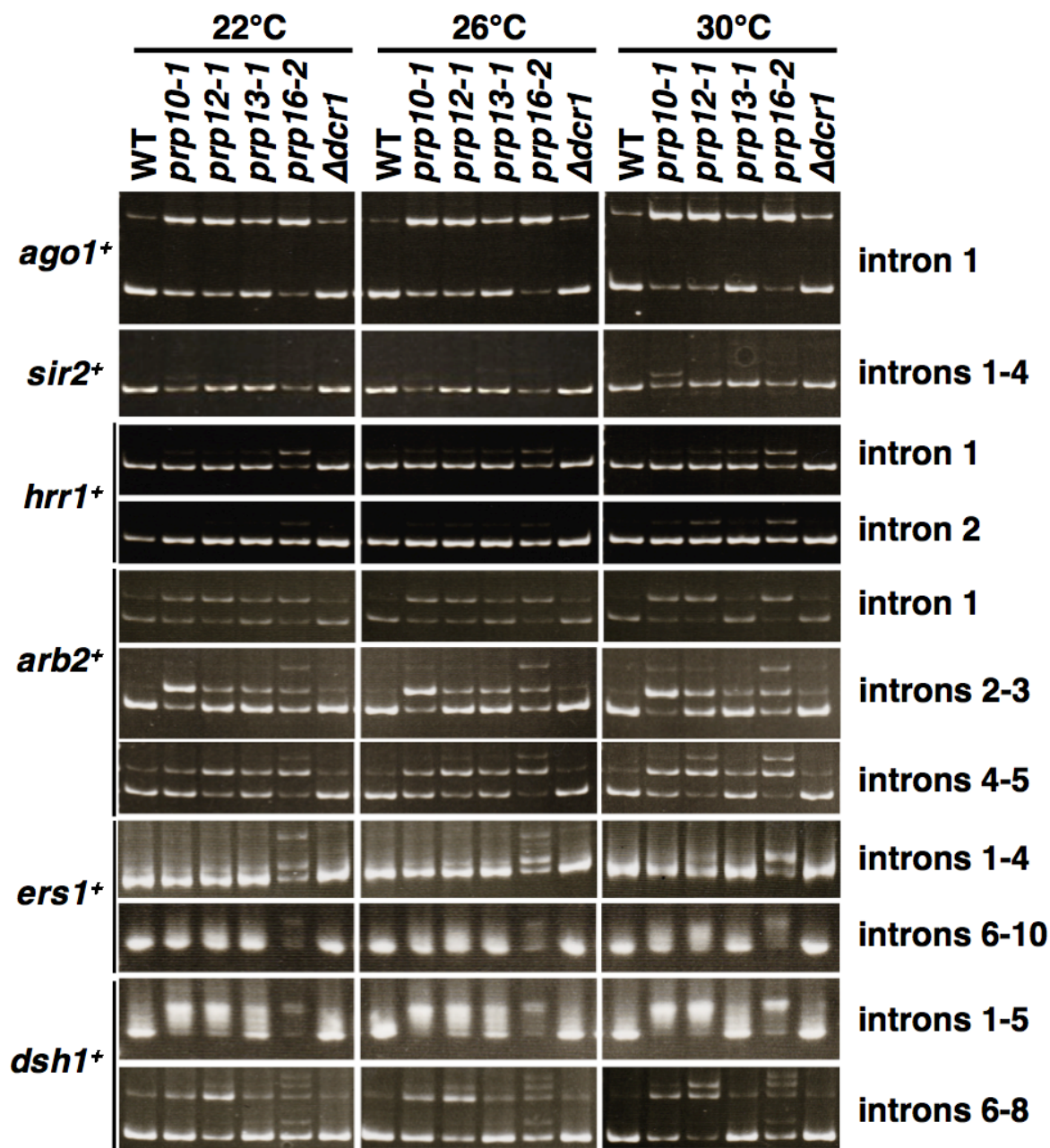

Supplement: S3 Fig — Total RNAs were isolated from the indicated strains cultured at 22°C, 26°C, or 30°C and then subjected to RT-PCR analysis using primers specific for the indicated intron regions. (PDF) [file pgen.1006606.s004.pdf]

S4 Figure

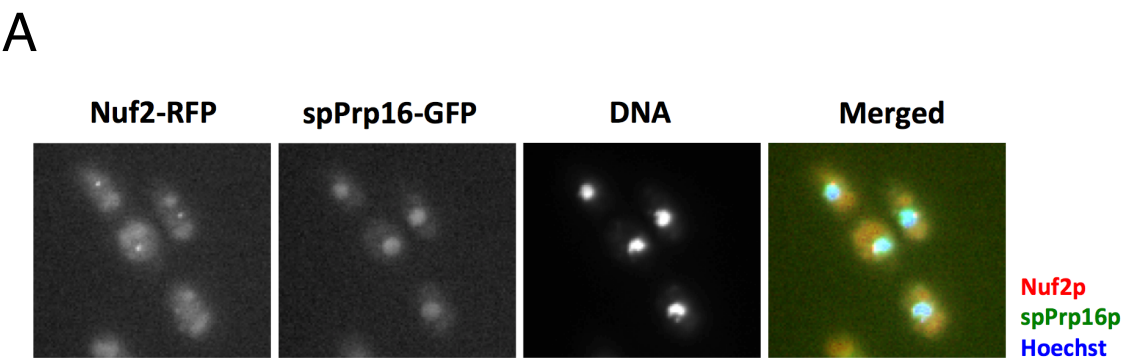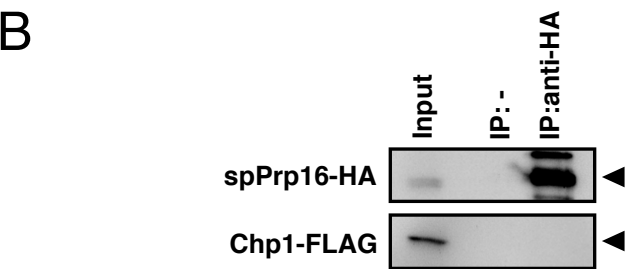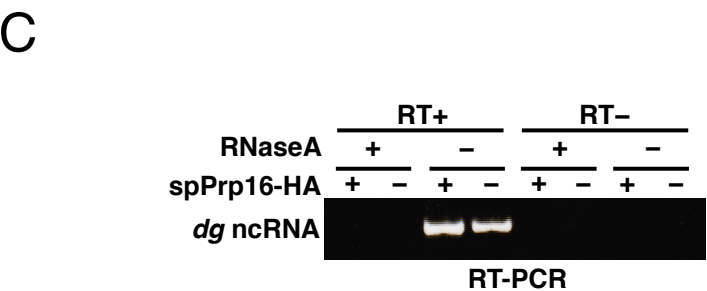

Supplement: S4 Fig — (A) spPrp16p-GFP expressed from the endogenous locus localized diffusely in the nucleus. No dot-like signals were observed. Cells cultured at 30°C were counterstained with Hoechst 33342 and observed through a Nikon Eclipse Ti fluorescence microscope equipped with an ORCA-R2 cooled CCD camera. (B) spPrp16-HA does not associate with Chp1p-FLAG. Co-immunoprecipitation analysis was carried out using a strain expressing spPrp16-HA and Chp1p-FLAG. spPrp16-HA was immunoprecipitated with (IP: anti-HA) or without (IP: -) an anti-HA antibody. The precipitates were then subjected to western blot analysis using anti-FLAG antibody. No band was detected in the precipitates with the anti-HA antibody, suggesting no association between spPrp16p and Chp1p. Arrowheads indicate positions of spPrp16-HA and Chp1p-FLAG. (C) Treatment of extracts with RNase A degraded dg ncRNA. Extracts from cells with (spPrp16-HA: +) or without (spPrp16-HA: -) a plasmid expressing spPrp16-HA were treated with 10 μg/ml RNase A at 37°C for 10 min (RNase A: +), and then subjected to the RT-PCR analysis. “RT-” indicates a reaction without reverse transcriptase. (PDF) [file pgen.1006606.s005.pdf]

S5 Figure

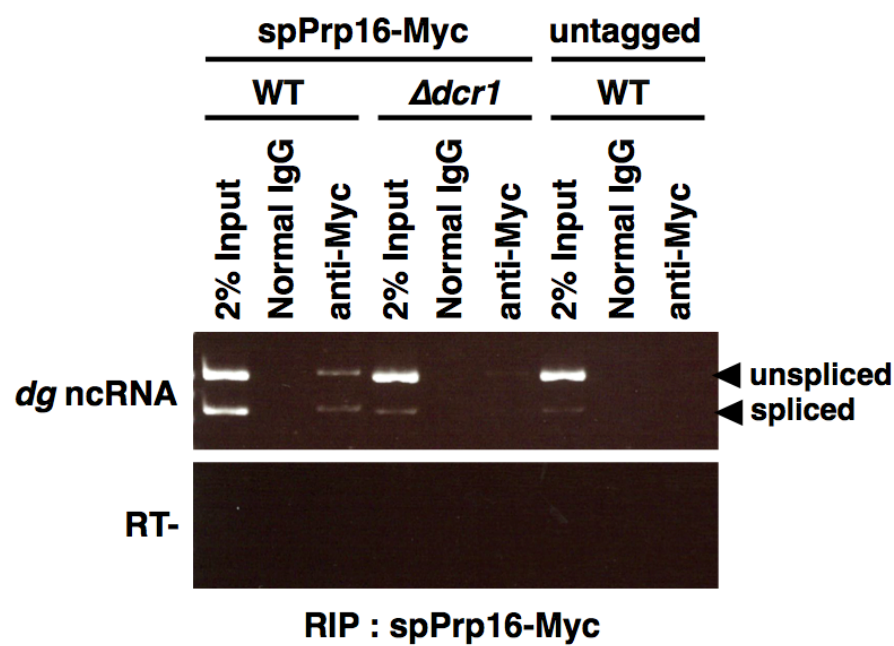

Supplement: S5 Fig — RT-PCR products prepared from the indicated precipitates were electrophoresed on an 8% polyacrylamide gel and stained with ethidium bromide. RNA immunoprecipitation was performed using anti-Myc antibody. “RT-” indicates a reaction without reverse transcriptase. (PDF) [file pgen.1006606.s006.pdf]

S6 Figure

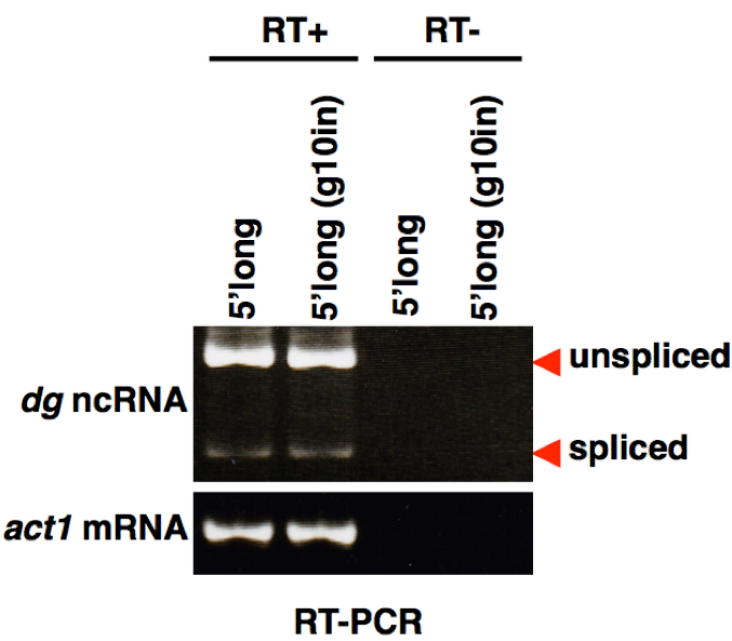

Supplement: S6 Fig — RT-PCR was performed using plasmid-specific primers. Act1 mRNA was detected as a loading control (act1 mRNA). “RT-” indicates a reaction without reverse transcriptase. (PDF) [file pgen.1006606.s007.pdf]

S7 Figure

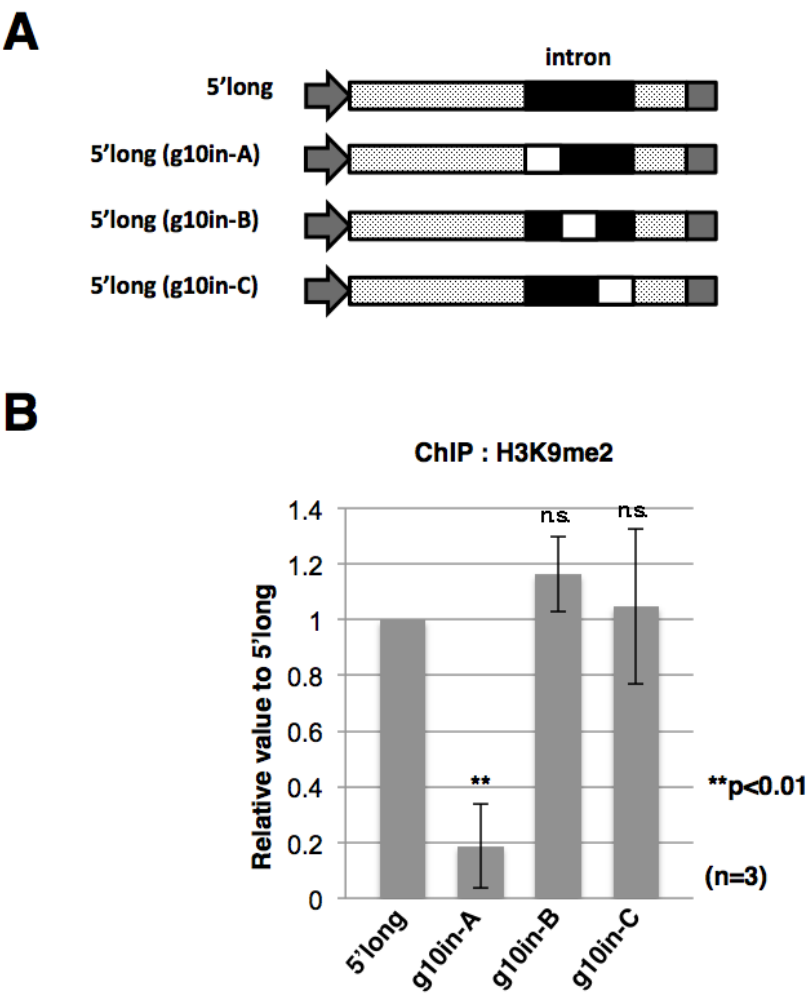

Supplement: S7 Fig — (A) Schematic representation of the chimeric 5’-long constructs, in which part of the intron was replaced with the sequence of the gcd10 intron (white boxes). (B) ChIP analysis of H3K9me2 on the chimeric 5’-long constructs. The 5’-long (g10in-A) construct in which the 5’ region of the dg intron was replaced with the corresponding region of the gcd10 intron had a reduced level of H3K9me2. (PDF) [file pgen.1006606.s008.pdf]

S8 Figure

A

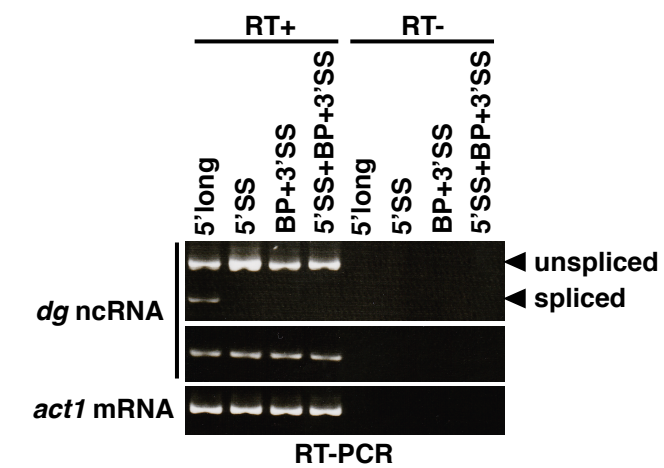

B

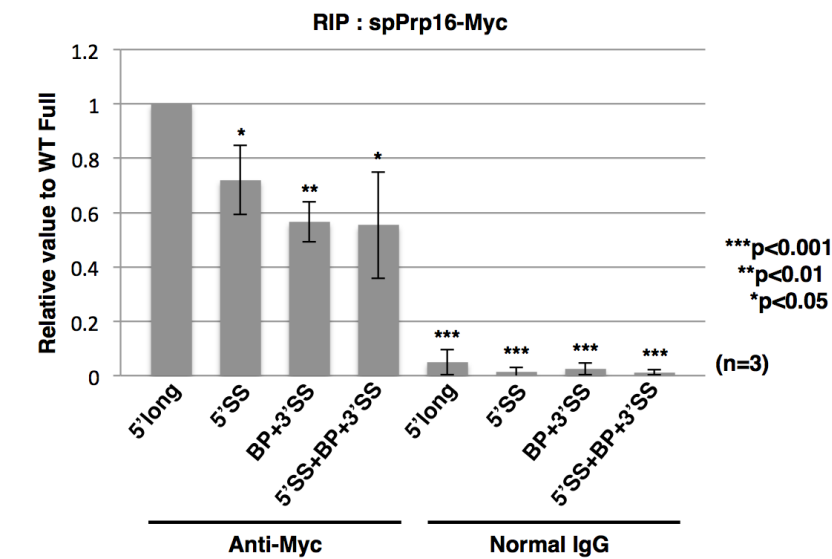

Supplement: S8 Fig — (A) Splicing of the dg ncRNA was completely inhibited by the mutations introduced. RT was performed using plasmid-specific primers. PCR was conducted using primers that amplified the region spanning the intron (upper panel), which were used for evaluation of the splicing reaction, or primers that amplified the region downstream of the intron (middle panel), which were used to determine the amounts of the transcripts. act1 mRNA was analyzed as a loading control (bottom panel). “RT-” indicates a reaction without reverse transcriptase. (B) RIP analysis of spPrp16-Myc was performed with the anti-Myc antibody (anti-Myc) or non-immune IgG (normal IgG) and strains possessing plasmids with the indicated mutations. qPCR was performed using plasmid-specific primers. (PDF) [file pgen.1006606.s009.pdf]

S9 Figure

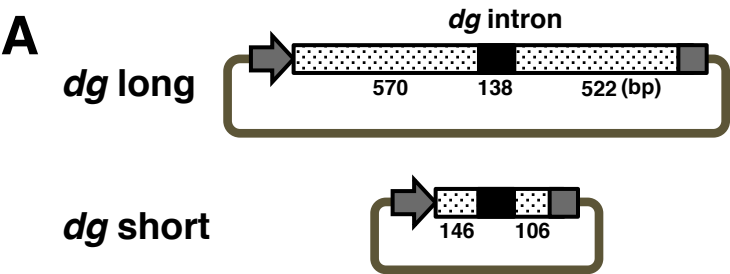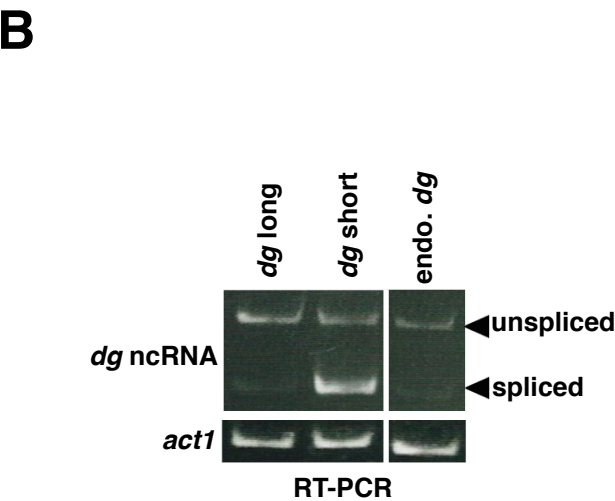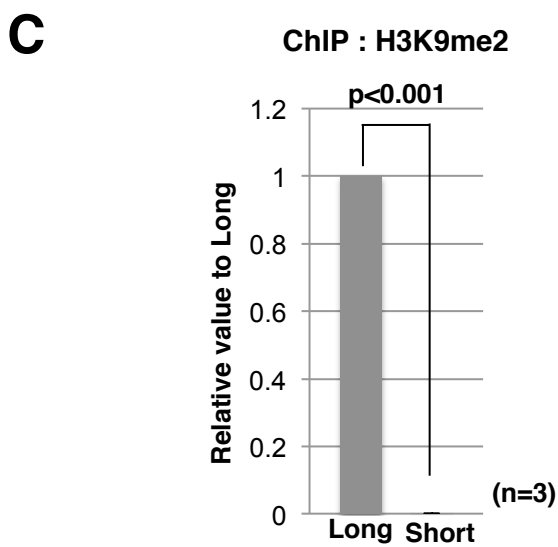

Supplement: S9 Fig — (A) Schematic representation of plasmids expressing dg transcripts. The dg-long and dg-short constructs contain the 1,230 and 390 bp fragments including the dg intron, in addition to the nmt1 promoter and terminator (gray arrow and box, respectively). (B) Splicing of dg ncRNAs expressed from plasmids or the endogenous locus was analyzed by RT-PCR using plasmid-specific primers (dg-long or dg-short) or endogenous gene-specific primers (endo. dg). The arrowheads indicate unspliced and spliced products. The dg-short transcript was spliced efficiently. No bands were detected in any samples without the reverse transcription reaction. act1 was detected as a loading control. (C) ChIP analysis of H3K9me2 at the dg region was performed using the H3K9me2 antibody and strains harboring the dg-long or dg-short plasmid. qPCR was performed using plasmid-specific primers. (PDF) [file pgen.1006606.s010.pdf]

S10 Figure

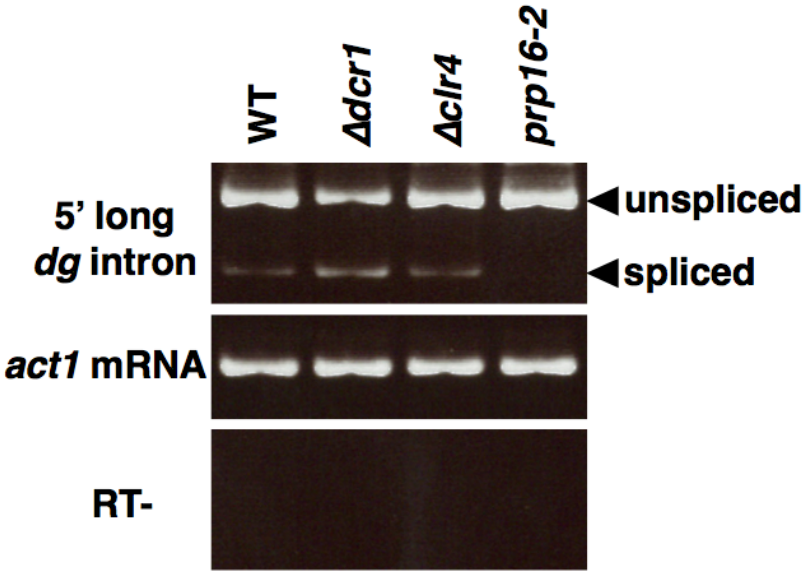

Supplement: S10 Fig — Splicing of the 5’-long dg intron in the indicated strains was analyzed by RT-PCR. Act1 mRNA was detected as a loading control (act1 mRNA). “RT-” indicates a reaction without reverse transcriptase. (PDF) [file pgen.1006606.s011.pdf]

S11 Figure

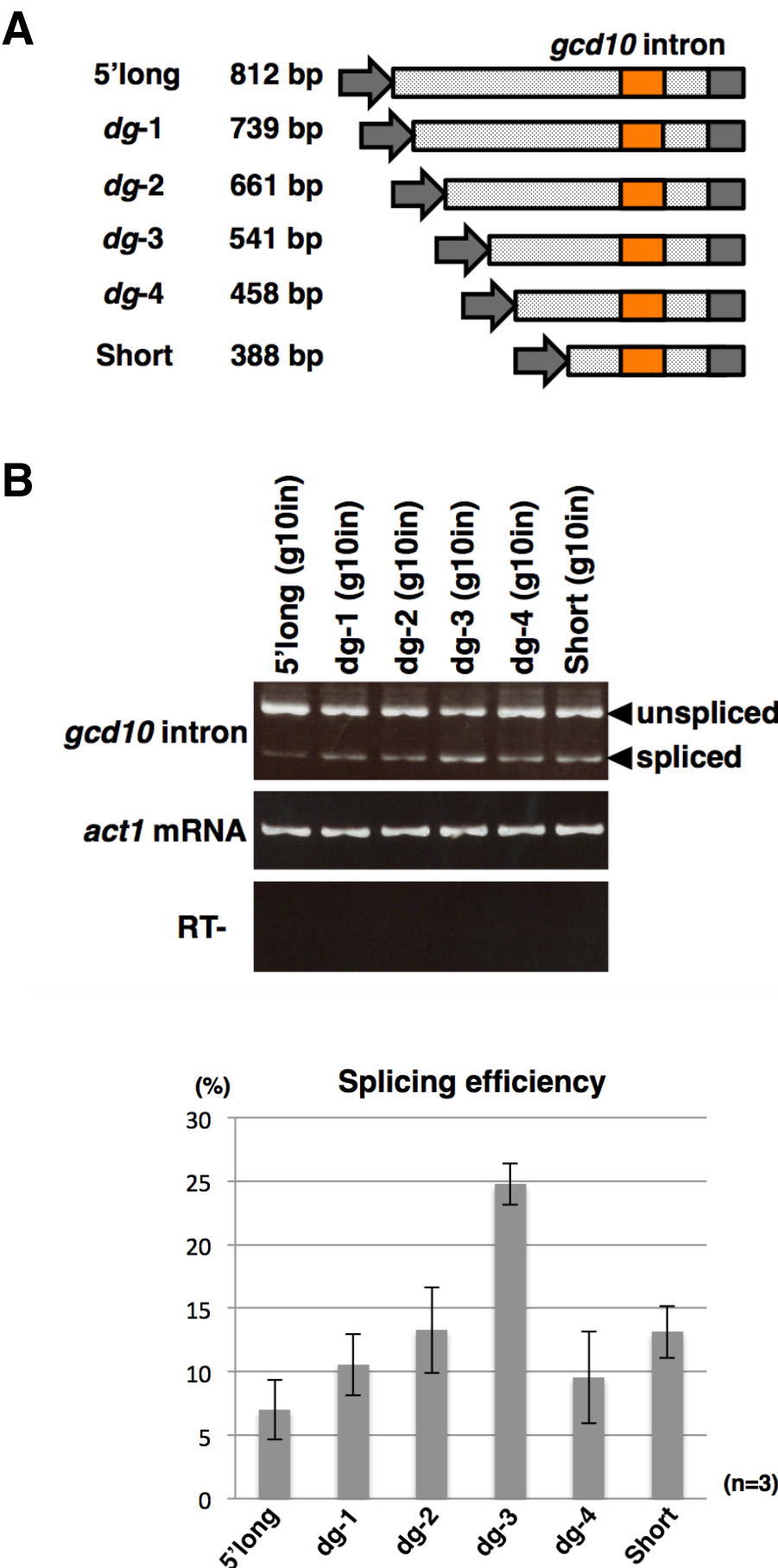

Supplement: S11 Fig — (A) Schematic representation of chimeric fragments containing the gcd10 intron and serially deleted upstream region. Constructs were fused to the nmt1 promoter and terminator (gray arrows and boxes). The numbers on the left denote the length of each intron-containing dg DNA fragment inserted between the nmt1 promoter and terminator. (B) Splicing of the gcd10 chimeric RNAs transcribed from serially deleted fragments was examined by RT-PCR (upper panel) and qRT-PCR (lower panel) using plasmid-specific primers. To draw a graph of splicing efficiency, the ratios of the spliced products to the total transcripts were calculated. In RT-PCR, Act1 mRNA was analyzed as a loading control. “RT-” indicates a reaction without reverse transcriptase. (PDF) [file pgen.1006606.s012.pdf]

S12 Figure

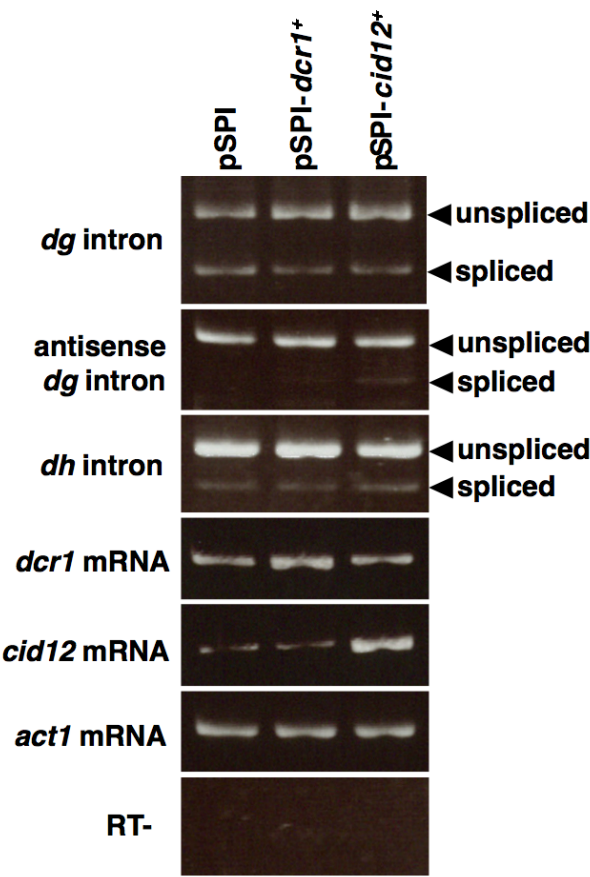

Supplement: S12 Fig — A multicopy plasmid (pSP1) harboring the dcr1+ or cid12+ gene was introduced into wild-type cells, and splicing of dg, antisense dg and dh ncRNAs was analyzed by RT-PCR (panels of dg intron, antisense dg intron and dh intron). Overexpression of the dcr1+ and cid12+ genes was confirmed by RT-PCR (panels of dcr1 mRNA and cid12 mRNA). Act1 mRNA was analyzed as a loading control (act1 mRNA). “RT-” indicates a reaction without reverse transcriptase. (PDF) [file pgen.1006606.s013.pdf]

S13 Figure

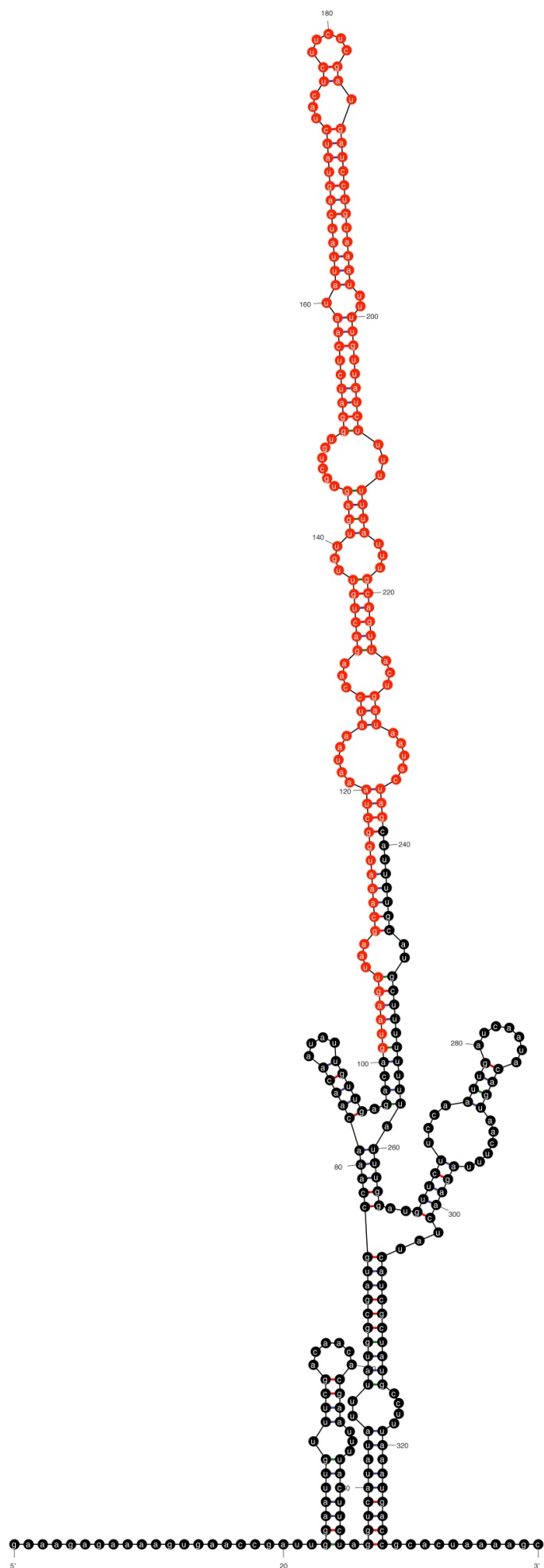

Supplement: S13 Fig — Secondary structure of the dg intron with upstream (-100 bp from the dg intron) and downstream (+100 bp from the dg intron) sequences was predicted using Mfold (http://unafold.rna.albany.edu/?q=mfold/RNA-Folding-Form). Mutations introduced in the splice sites or branch point shown in Fig 7 did not affect the predicted structure significantly. Because this region is included in the dg-short construct, deletions of the upstream sequence shown in Fig 8A also do not affect the structural prediction. The intron region is indicated in red. (PDF) [file pgen.1006606.s014.pdf]

S14 Figure

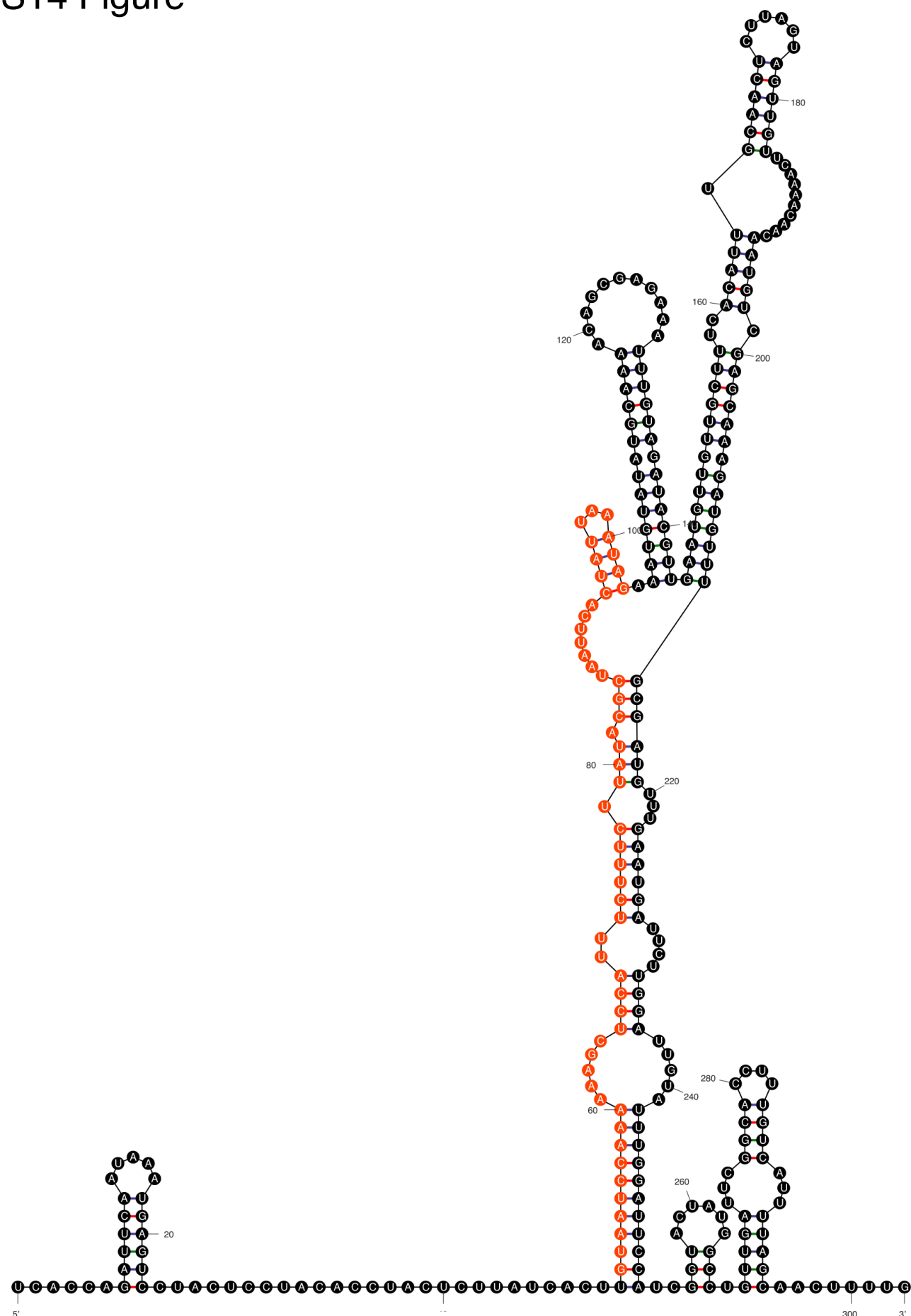

Supplement: S14 Fig — Secondary structure of the dh intron with upstream (-50 bp from the dh intron) and downstream (+200 bp from the dh intron) sequences was predicted using Mfold (http://unafold.rna.albany.edu/?q=mfold/RNA-Folding-Form). The intron region is indicated in red. (PDF) [file pgen.1006606.s015.pdf]
