# Supplementary material for: The intron in centromeric noncoding RNA facilitates RNAi-mediated formation of heterochromatin
Source: PLoS Genet. 2017 Feb 23;13(2):e1006606. doi: 10.1371/journal.pgen.1006606 (PMC5322907; doi:10.1371/journal.pgen.1006606)
Supplement: S1 Table — (PDF) [file pgen.1006606.s016.pdf]

S1 Table Strains used in this study

Strain	Genotype
972	*h*⁻
HM123	*h⁻ leu1-32*
UR470	*h⁺ leu1-32 ura4-D18*
UR471	*h⁻ leu1-32 ura4-D18*
UR390	*h⁻ prp16-1*
prp16-2	*h⁻ leu1-32 prp16-2*
Δprp16	*prp16::KanMX6 ade6-210 leu1-32 ura4-D18*
SP587	*h⁺ leu1-32 ura4-D18 ago1::KanR*
SP473	*h⁺ leu1-32 ura4-D18 dcr1::KanR*
FY648	*h⁺ otr1R(dg-glu BamHI-SpeI frag.)SphI::ura4⁺ oriI leu1-32 ura4-DS/E ade6-210*
MR2	*prp16-2 otr1R (dg-glu BamHI-SpeI frag.) SphI::ura4+*
FY711	*h⁹⁰ swi6-115 otr1R(dg-glu BamHI-SpeI frag.)SphI::ura4⁺ oriI leu1-32 ura4-DS/E ade6-210*
FY15720	*h⁺ his2 ura4-D18 leu1-32 nuf2+::mRFP-ura4+*
Cid12-TAP	*h⁺ leu1-32 ura-DS/E ade6-216 imr1R::ura4 Cid12-TAP-hph rdp1::nat*
WT Cid12	*leu1-32 ura- ade6-704 Cid12-5FLAG-KanMX6*
Δdcr1 Cid12	*leu1-32 ura- dcr1::KanR Cid12-5FLAG-KanMX6*
Δclr4 Cid12	*leu1-32 ura- clr4::ura4 Cid12-5FLAG-KanMX6*
Δrdp1 Cid12	*leu1-32 ura- rdp1::nat Cid12-5FLAG-KanMX6*
WT Prp16	*leu1-32 ura- ade6-704 Prp16-Myc*
Δdcr1 Prp16	*leu1-32 ura- dcr1::KanR Prp16-Myc*
BG_H3433	*h⁺ leu1-32 ura4-D18 ade6-M216 cid12 orfΔ::KanMX4*
FY14209	*h⁻ leu1-32 ura4-D18 clr4::ura4⁺*
UR230	*h⁻ prp10-1*
SU100-2A	*h⁻ prp12-1*
prp13-1	*h⁺ leu1-32 prp13-1*
SPT14	*h⁺ leu1-32 ura4-DS/E ade6-M210 otr::ura4⁺ Chp1-FLAG*
