# Supplementary material for: The intron in centromeric noncoding RNA facilitates RNAi-mediated formation of heterochromatin
Source: PLoS Genet. 2017 Feb 23;13(2):e1006606. doi: 10.1371/journal.pgen.1006606 (PMC5322907; doi:10.1371/journal.pgen.1006606)
Supplement: S2 Table — (PDF) [file pgen.1006606.s017.pdf]

S2 Table Plasmids used in this study

pSP1-*prp16*$^{+}$
pRE-Prp16-GFP
pRE-EGFP
pRE-Prp16-3FLAG
pRE-3FLAG
pRE-Prp16-HA
pcc2-KpnI-Full + Leu2 (Minichromosome, Full)
pcc2-KpnI-Intron Less + Leu2 (Minichromosome, Less)
pREP1-dg long
pREP1-5'long
pREP1-dg-1
pREP1-dg-2
pREP1-dg-3
pREP1-dg-4
pREP1-Short
pREP1-5'long (g10in)
pREP1-5'long (g10in-A)
pREP1-5'long (g10in-B)
pREP1-5'long (g10in-C)
pREP1-5'SS
pREP1-BP+3'SS
pREP1-5'SS+BP+3'SS
pREP1-Act1/dg
pREP1-dg-1 (g10in)
pREP1-dg-2 (g10in)
pREP1-dg-3 (g10in)
pREP1-dg-4 (g10in)
pREP1-Short (g10in)
pREP1-dg+gcd10
pSP1-Sau3AI-Sau3AI (1)
pSP1-VspI-SacI (2)
pSP1-Sau3AI-VspI (3)
pSP1-dcr1^{+}
pSP1-cid12^{+}
