# Supplementary material for: The intron in centromeric noncoding RNA facilitates RNAi-mediated formation of heterochromatin
Source: PLoS Genet. 2017 Feb 23;13(2):e1006606. doi: 10.1371/journal.pgen.1006606 (PMC5322907; doi:10.1371/journal.pgen.1006606)
Supplement: S3 Table — (PDF) [file pgen.1006606.s018.pdf]

## S3 Table Primers used in this study

| Primer | Sequence | |
|---|---|---|
| | **Forward (5'→3')** | **Reverse (5'→3')** |
| ura4 | GAGGGGATGAAAAATCCCAT | TTCGACAACAGGATTACGACC |
| dg (qPCR) | TAGAACTATCATCGCTATGC | AGAGAGTATAGAGAGAGGCTG |
| dh | CTACGCTTGATTTGAGGAAGG | AAAGTATGAGTCGCAGAAGTG |
| act1 | GGCATCACACTTTCTACAACG | GAGTCCAAGACGATACCAGTG |
| minichromosome (intron) | GATGCCAAACAACAATATTG | CGCAATTAATGTGAGTTAGC |
| minichromosome (qPCR) | TAGAACTATCATCGCTATGC | CGCAATTAATGTGAGTTAGC |
| dg (intron) | TCCATCCGCAGTTGGGAG | TACCATGCTTTTAGTGCGG |
| plasmid specific (qPCR) | TAGAACTATCATCGCTATGC | AACCCTAGCAGTACTGGC |
| spliced dg ncRNA (plasmid) | CAACAATATTGTTGAGACACA | AACCCTAGCAGTACTGGC |
| unspliced dg ncRNA (plasmid) | CAGTATCTACTCTTCTCGATG | AACCCTAGCAGTACTGGC |
| total dg ncRNA (plasmid) | TAGAACTATCATCGCTATGC | AACCCTAGCAGTACTGGC |
| ago1 (intron 1) | CCTAACGAGACTATCAAC | CTTGATGGTGCCATCAGC |
| sir2 (introns 1-4) | TATGTGCGGCAGTAAAG | TCTCCTAGCAACCTCACG |
| hrr1 (intron 1) | GGAATATACTCGGTTGAC | CTAACATCTGAGCTTCG |
| hrr1 (intron 2) | AATGACCGGACAGAGGTC | GTGATCTCCTAGACCTTG |
| arb2 (intron 1) | AGTTACCAAAGATAAAGAGTACAACGA | TGTGGTTCTGACGGTGAAGA |
| arb2 (introns 2-3) | CCCTCTGCGCTATTATGGGA | AGTGCTCACTGAATTGGACA |
| arb2 (introns 4-5) | TGAAGAGCTTCATGACCCCG | TCTCAAGCAAAACATCGCCA |
| ers1 (introns 1-4) | GCATGTACCATCTGTAAGCTCG | TTCCTTCCCATTCAAAAGGAGT |
| ers1 (introns 6-10) | CGGTGTGGGAAGCTTATACT | TGTCGAGACAAACGAAGGCT |
| dsh1 (introns 1-5) | ATGGCAGAAAATAAGAAATTTTCAATAAGAAAAAAAC | ACATTAAATAAAGATGCTGAAACGAAA |
| dsh1 (introns 6-8) | CCTAGCGGGTGGTATCTTGA | TCGTGTGCTTCAAACCGAGT |
| antisense dg intron | ACTGCTTATCTTTTGCAACC | CTCTTGTGCTCAGGCTGG |
| dh intron | ACATGGCTTAGTTTCACAC | GCTCGACATTGTTGTTTTG |
| dcr1 | CCCTTACTCCTCGTATATAG | AGTCTCCCTGAACGCTTC |
| cid12 | AAGTACTTGGATGCTGATGC | GACTCCCCAAAAATAGAAG |
